# High-throughput amplicon sequencing and stream benthic bacteria: identifying the best taxonomic level for multiple-stressor research

**DOI:** 10.1038/srep44657

**Published:** 2017-03-22

**Authors:** R. K. Salis, A. Bruder, J. J. Piggott, T. C. Summerfield, C. D. Matthaei

**Affiliations:** 1 Department of Zoology, University of Otago, Dunedin, New Zealand; 2 School of Natural Sciences, Trinity College Dublin, the University of Dublin, Dublin 2, Ireland; 3 Department of Botany, University of Otago, Dunedin, New Zealand

## Abstract

Disentangling the individual and interactive effects of multiple stressors on microbial communities is a key challenge to our understanding and management of ecosystems. Advances in molecular techniques allow studying microbial communities *in situ* and with high taxonomic resolution. However, the taxonomic level which provides the best trade-off between our ability to detect multiple-stressor effects versus the goal of studying entire communities remains unknown. We used outdoor mesocosms simulating small streams to investigate the effects of four agricultural stressors (nutrient enrichment, the nitrification inhibitor dicyandiamide (DCD), fine sediment and flow velocity reduction) on stream bacteria (phyla, orders, genera, and species represented by Operational Taxonomic Units with 97% sequence similarity). Community composition was assessed using amplicon sequencing (16S rRNA gene, V3-V4 region). DCD was the most pervasive stressor, affecting evenness and most abundant taxa, followed by sediment and flow velocity. Stressor pervasiveness was similar across taxonomic levels and lower levels did not perform better in detecting stressor effects. Community coverage decreased from 96% of all sequences for abundant phyla to 28% for species. Order-level responses were generally representative of responses of corresponding genera and species, suggesting that this level may represent the best compromise between stressor sensitivity and coverage of bacterial communities.

Most ecosystems are being affected by multiple stressors which can interact, causing responses that are larger or smaller than predicted from the sum of the individual stressor effects^1^,^2^. Multiple-stressor effects can exacerbate degradation of ecosystems. For example, multiple stressors contribute to the impairment of coral reefs^3^ and marine organisms show complex and often synergistic responses to simultaneous ocean acidification and warming^4^. Interacting multiple stressors have also been identified as an important driver of freshwater biodiversity loss^5^, which is disproportionally high compared to marine or terrestrial ecosystems. Addressing these global environmental issues requires understanding interactive stressor effects on structure and functioning of affected ecosystems. Bacteria play a major but understudied role in freshwater ecosystems, driving nutrient cycling, metabolic processes and many other biogeochemical processes and ecosystem functions^6^,^7^,^8^.

Agricultural intensification is one of the greatest threats to freshwater biodiversity^9^,^10^,^11^. Agricultural stressors affecting stream ecosystems include elevated inputs of nutrients, other agricultural chemicals and fine sediment, but also reductions in discharge and current velocity due to water abstraction for irrigation^12^,^13^,^14^,^15^. Intensively farmed pastures receive high inputs of nutrients (particularly nitrogen and phosphorus) through animal urine, faeces and fertilisers^9^,^16^. High nitrogen concentrations in farmland soils can leach into waterways^9^, where excess nutrient can increase stream bacterial biomass^17^,^18^,^19^ and affect bacterial community structure^20^,^21^,^22^. Nitrogen losses from farmland soils can be reduced by application of nitrification inhibitors, for example dicyandiamide (DCD)^23^,^24^,^25^. Earlier studies indicate that DCD impairs the activity of ammonium-oxidising soil bacteria by restricting their uptake of ammonium while having minimal effect on non-target bacteria^26^,^27^. However, DCD itself is water soluble, readily leached from soils^28^ and can disrupt nitrification in freshwater ecosystems^29^. Nevertheless, the effects of nitrification inhibitor leaching on bacterial communities in agricultural streams are still unknown. Fine sediment can also influence stream biofilms, by increasing turbidity and transforming the substratum, for instance by smothering existing biofilms and increasing substratum instability^9^,^30^,^31^. Water abstraction for irrigation may likewise affect bacterial communities. For example, reductions in flow velocity increase substratum boundary layer thickness, thereby reducing exchange of nutrients and oxygen between water column and biofilm^32^,^33^,^34^,^35^.

Until recently, microbial ecologists have lacked methodological tools for *in-situ* assessments of microbial communities. Traditional investigations relied on information gained through cultivation, restricting knowledge to the small proportion of microbes that can be cultured^36^. Recent advances in molecular techniques allow studying microbial communities directly in their environment^37^. Techniques such as high-throughput amplicon sequencing provide taxonomic resolution down to the level of Operational Taxonomic Units (OTUs), which are widely considered to be equivalent to the species level when clustered at 97% similarity^38^,^39^,^40^, and permit in-depth analyses of changes in community composition and phylogenetic diversity. However, few studies have used these techniques to investigate multiple-stressor effects on freshwater bacteria, and manipulative experiments that allow developing mechanistic understanding are lacking. Moreover, it is unknown which level of taxonomic resolution provides the best trade-off between maximising our ability to detect and interpret multiple-stressor effects versus the goal of studying the entire bacterial community. Because higher taxonomic levels are more likely to include taxa with opposing responses, one may expect lower taxonomic levels to respond more sensitively to stressors, but this hypothesis remains untested.

To address these knowledge gaps, we used 64 outdoor mesocosms (simulating small agricultural streams) to investigate the individual and combined effects of four agricultural stressors (nutrient enrichment, DCD leaching, sedimentation and flow velocity reduction) on stream benthic bacteria at four taxonomic levels (phylum, order, genus and species: OTU with clustering at 97% sequence similarity). These mesocosms, which have been used to investigate multiple-stressor effects on stream periphyton, invertebrates and organic matter decomposition^35^,^41^,^42^,^43^, permit studying stressor effects under tightly controlled, statistically powerful yet highly realistic ecological conditions. They allow natural immigration and emigration of stream biota and experience the same environmental conditions as the river feeding them, a key strength of our study compared to laboratory experiments. We hypothesised that:

- The four stressors interactively affect the bacterial community; more specifically, nutrient enrichment changes bacterial community composition (via positive effects on nutrient-limited taxa), DCD addition disadvantages the target ammonium-oxidising bacteria while having little effect on non-target bacteria, sediment addition and reduced flow velocity benefit some taxa while negatively affecting others;
- For both single-stressor effects and interactions, lower taxonomic levels (genus, species: OTU with clustering at 97% similarity) respond more sensitively than higher taxonomic levels (phylum, order) because the latter are more likely to include taxa with opposing responses to stressors, thus diluting the overall response.

## Results

The 16S rRNA dataset rarefied to 128,600 reads per sample clustered into 229,192 OTUs at a 97% level of sequence similarity (for 75,556 OTUs identity was unassigned). Resulting OTUs (species) were allocated to 1,385 genera within 758 families, 466 orders, 229 classes and 68 phyla.

### Community taxon richness and evenness

**Figure 1:**
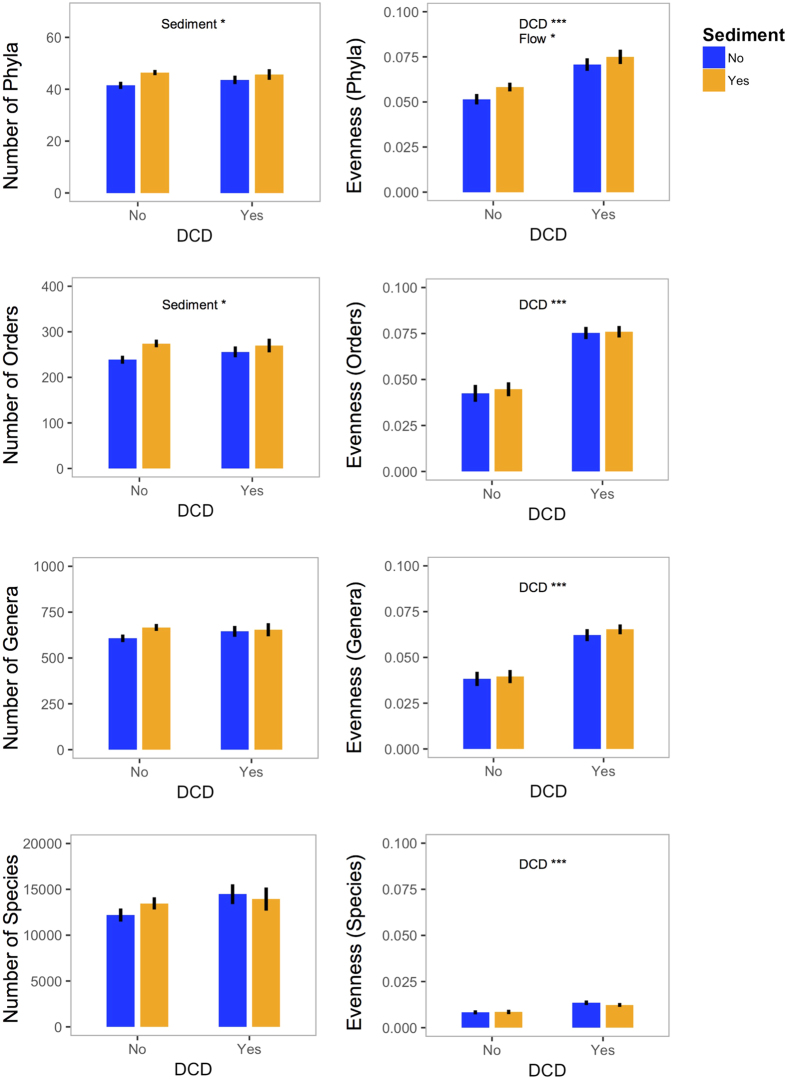
Mean taxon richness (left panels) and community evenness (right panels) at the phylum, order, genus and species levels in the DCD and sediment treatments (pooled for flow and nutrient treatments). Error bars represent standard errors. *P* < 0.001 = ***P < 0.01 = **P < 0.05 = *.

DCD addition increased bacterial community evenness at all four taxonomic levels (Fig. 1, Table S1). Evenness of phyla was also increased by flow velocity reduction but was uninfluenced by nutrients or sediment addition. Taxon richness at the phylum and order levels was increased by sediment addition but unaffected by DCD, nutrients or flow reduction.

### Abundant phyla

**Figure 2:**
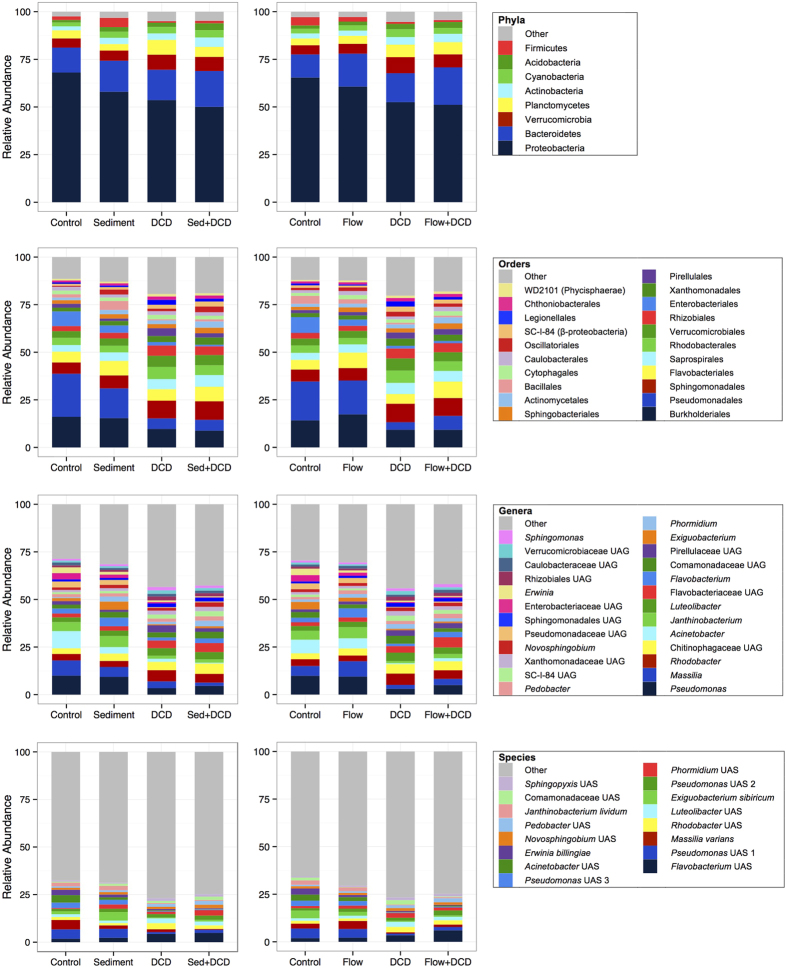
Mean relative abundances of the abundant phyla, orders, genera and species in the DCD and sediment treatments (left panels) and in the DCD and flow velocity treatments (right panels).

At the phylum level, Proteobacteria dominated (57.5% of all sequences), followed by Bacteroidetes (16.0%) and Verrucomicrobia (6.3%). Overall, community composition of the eight abundant phyla was affected by DCD, sediment, flow velocity and a DCD × sediment interaction, but not by nutrients (multivariate MANOVA results, Table S1, Fig. 2). DCD and sediment were the most pervasive stressors, each affecting six phyla, with average effect sizes (ES, for significant effects) of 0.34 and 0.22, respectively (Table 1). Two phyla responded to flow velocity (average ES = 0.16) and none to nutrients. Relative abundances of Proteobacteria and Firmicutes decreased with DCD addition, whereas those of Verrucomicrobia, Planctomycetes, Actinobacteria and Acidobacteria increased. Proteobacteria and Planctomycetes decreased with sediment addition while four phyla increased (Bacteroidetes, Actinobacteria, Acidobacteria, Firmicutes). Proteobacteria also decreased under reduced flow velocity whereas Bacteroidetes increased. Two of the eight phyla showed stressor interactions (Table S1, Figure S1), i.e. Proteobacteria a DCD × sediment and Verrucomicrobia a DCD × flow interaction (see Supplementary Information for details).

### Abundant orders

The most abundant orders were Burkholderiales (Betaproteobacteria, contributing 12.6% of all sequences), Pseudomonadales (Gammaproteobacteria, 12.4%), Sphingomonadales (Alphaproteobacteria, 8.0%) and Flavobacteriales (Bacteroidetes, 6.7%) (Fig. 2). Composition of the 21 abundant orders was affected by all four stressors and DCD × sediment, DCD × nutrients and sediment × flow interactions (multivariate results, Table S1). DCD was the most pervasive stressor, affecting 16 orders (mean ES = 0.34), with four responding negatively and 12 positively (see Table S1 for details). Flow velocity reduction affected nine orders (ES = 0.17), with four responding negatively and five positively. Sediment addition also affected nine orders (ES = 0.16), with three becoming less prevalent while six increased. Only one order (Cytophagales) responded to nutrients (ES = 0.20), becoming rarer with enrichment. Six of the 21 orders showed one or two 2-way interactions each (Table S1, Figure S2), for DCD × flow (three orders), DCD × sediment (two) interactions, sediment × flow and flow × nutrients (one each).

### Abundant genera

The most numerous genera were *Pseudomonas* (6.9% of all sequences), *Massilia* (4.7%), *Rhodobacter* (4.2%), and an unidentified Chitinophagaceae genus (4.1%). Composition of the 25 abundant genera was affected by DCD, sediment, flow velocity and a DCD × flow interaction (multivariate results, Table S1). Again DCD was the most pervasive stressor, affecting 19 genera (mean ES = 0.32), with nine responding negatively and ten positively (see Table S1 for details). Sediment affected 12 genera (mean ES = 0.17; 5 negatively, 7 positively) and flow velocity reduction 9 genera (ES = 0.16; 6 negatively, 3 positively), whereas nutrients affected none. Five of the 25 genera showed stressor interactions (Table S1, Figure S3), for DCD × flow (three genera), DCD × sediment and sediment × nutrients (one each).

### Abundant and common species

Composition of the 16 abundant species (each contributing 1.0–3.4% of all sequences) was affected by DCD, sediment, flow, DCD × sediment and DCD × flow interactions, but not by nutrients (multivariate results, Table S1). Once more DCD was the most pervasive stressor, affecting 14 species (mean ES = 0.27), followed by sediment (8 species, ES = 0.16) and flow velocity (3 species, ES = 0.28), whereas nutrients had no significant effects. When the 27 common species (defined here as 0.3–1.0% of all sequences; see Methods) were included in the MANOVA, only sediment and flow affected bacterial community composition (43 species) significantly (*P* = 0.05 for DCD). Nevertheless, DCD was still the most pervasive stressor based on the univariate effects of the MANOVA, affecting a further 24 common species (mean ES = 0.27), followed by sediment (18 additional species, ES = 0.20), flow velocity (10 additional species, ES = 0.24) and nutrients (1 species, ES = 0.37).

**Figure 3:**
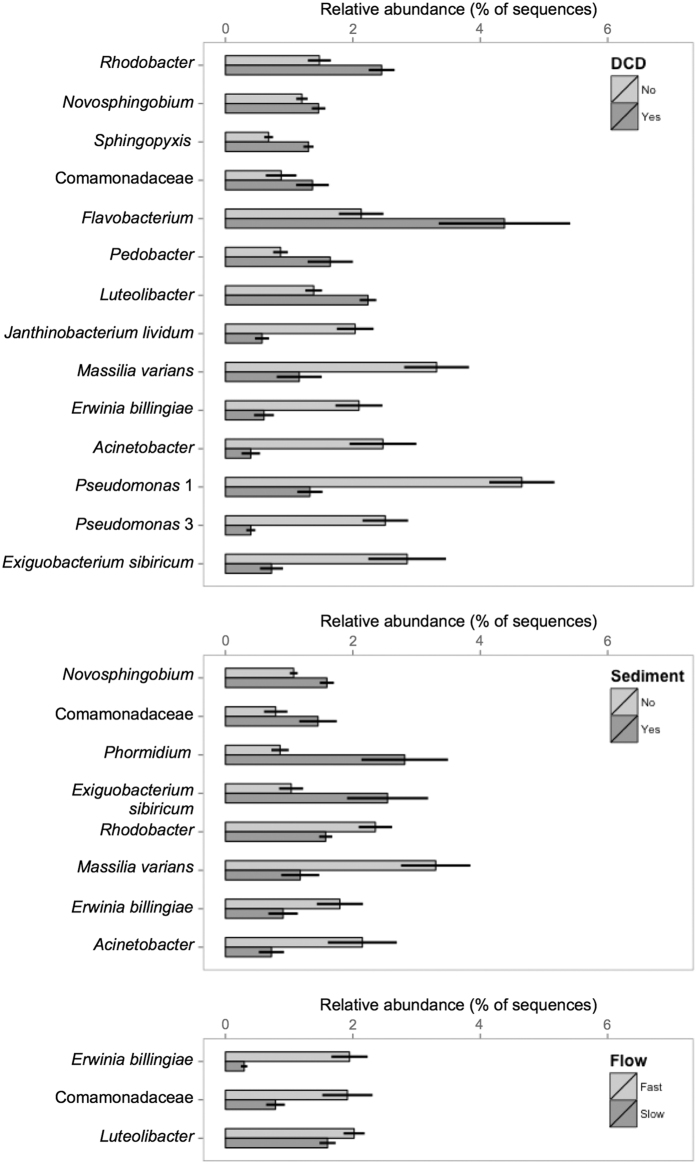
Mean relative abundances (±SE) of the abundant species that showed significant responses to the DCD (upper panel), sediment (middle panel) or flow velocity treatments (lower panel). For complete species names see Table S1.

Seven abundant species responded positively to DCD (*Rhodobacter, Novosphingobium, Spingopyxis*, Comamonadaceae, *Flavobacterium, Pedobacter* and *Luteolibacter* species), whereas another seven responded negatively (*Janthinobacterium lividum, Massilia varians, Erwinia billingiae*, an *Acinetobacter* species, two *Pseudomonas* species and *Exiguobacterium sibiricum*) (Fig. 3). Four species each responded negatively (*Exiguobacterium sibiricum* and *Novosphingobium*, Comamonadaceae and *Phormidium* species) or positively (*Massilia varians, Erwinia billingiae*, and *Rhodobacter* and *Acinetobacter* species) to sediment. All three species responding to flow (*Erwinia billingiae* and Comamonadaceae and *Luteolibacter* species) decreased with flow velocity reduction.

The common species showed similar responses to DCD, with an equal number (12 species) showing positive and negative responses, while eight responded positively to sediment and ten responded negatively to sediment (see Table S1 for details). Responses to flow velocity reduction were both positive (six species) and negative (four species), and one common species (a species of Enterobacteriaceae) showed a positive response to nutrient enrichment.

Three of the 16 abundant species showed stressor interactions, two DCD × flow interactions and one DCD × sediment interaction (Table S1, Figure S4). Of the 27 common species, ten showed one or two 2-way interactions, most being DCD × sediment and DCD × flow (four each), followed by DCD × nutrients (two species), and sediment × flow, sediment × nutrients and DCD × sediment × nutrients (one each) (Figures S5 and S6). One common species, *Kaistobacter* sp., showed a 3-way interaction (DCD × sediment × nutrients) (Figure S6).

### Comparison across taxonomic levels

Pervasiveness of single-stressor effects and their effect sizes varied little across taxonomic levels (Table 1). DCD increased community evenness at all taxonomic levels and affected around 75% of abundant phyla, orders and genera, but slightly more of the abundant species (88%), and this pattern remained unchanged when common species (0.3–1.0% of sequences) were included (88%). Sediment had its most pervasive effects on abundant phyla (75%, compared to 50% or less for abundant orders, genera and species, and 60% for abundant plus common species combined). Flow velocity effects were most frequent on orders (43%), followed by genera (36%), phyla (25%) and species (19%, this increased to 30% when common species were included). Nutrient effects were rare, occurring only for one abundant order and one common species. The largest number of interactive effects on abundant taxa was also detected on orders (29%, compared to 25% of phyla, 20% of genera, 19% of species). The percentage for species increased to 33% when common species were added.

**Figure 4:**
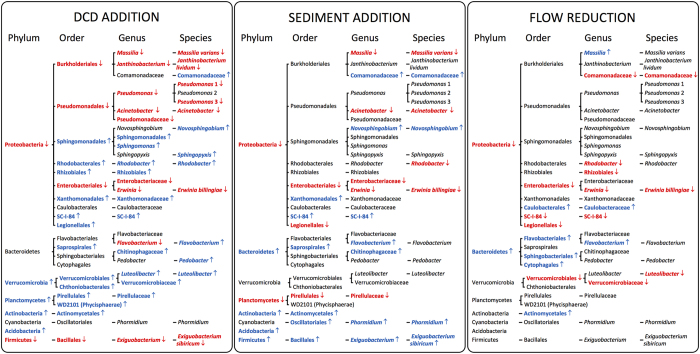
Directions of significant responses of the abundant (>1.0% of all sequences) phyla, orders, genera and species to DCD addition, sediment addition or flow velocity reduction. Arrows and colours indicate positive (↑, blue) and negative responses (↓, red). For complete taxa names see Table S1.

The abundant species, genera and orders within the phylum Firmicutes showed consistent negative responses to DCD (Fig. 4). The lower taxonomic levels within the phyla Verrumicrobia, Planctomycetes and Actinobacteria showed consistent positive responses to DCD. By contrast, lower taxonomic levels within the phyla Proteobacteria and Bacteriodetes showed mixed responses to DCD. Proteobacteria within the orders Sphingomonadales, Rhodobacterales, Rhizobiales, Xanthomonadales, the Beta-proteobacteria SC-1-84 and Legionellales all responded positively, whereas those within the orders Enterobacteriales, Pseudomonadales and Burkholderiales responded negatively, except for an unassigned species of the family Comamonadaceae in the genus Burkholderiales.

Responses to sediment were consistent across taxonomic levels for the abundant phyla Bacteroidetes, Planctomycetes, Actinobacteria and Firmicutes. For Cyanobacteria no significant response occurred at the phylum level but a consistent positive response was found for the abundant species and genus *Phormidium* within the order Oscillatoriales. While the phylum Proteobacteria responsed negatively to sediment, the abundant orders, genera and species within this phylum showed mixed responses. These were consistent either at the order (Enterobacteriales, SC-1-84) or genus level (*Massillia*, Comamonadaceae UAG, *Acinetobacter* and *Novosphingobium*).

Abundant orders and genera within the phylum Bacteroidetes all responded positively to flow velocity reduction, while those within the phylum Proteobacteria showed mixed responses to flow velocity reduction, with the response direction consistent at either the order or genus level. Since only one significant response to nutrients was found for abundant taxa, response directions could not be compared across taxonomic levels.

## Discussion

### Stressor main effects and interaction

We conducted the first full-factorial, 4-stressor field experiment employing amplicon sequencing to investigate individual and interactive effects of multiple stressors on freshwater bacteria. In our mesocosms mimicking small streams, common agricultural stressors had considerable effects on benthic bacterial communities (richness, evenness, community composition) and stressors interacted in several cases, supporting our first hypothesis. Of the four manipulated stressors, DCD was the most pervasive (in terms of size and prevalence of effects), significantly affecting community evenness and relative abundances of nearly all abundant taxa (>1% of all sequences, as defined by Baltar *et al*.^44^), regardless of taxonomic resolution. Fine sediment was the second-most pervasive stressor, followed by flow velocity reduction, whereas nutrient enrichment had surprisingly little effect.

The strong effects of DCD on the bacterial community did not support our prediction that DCD should have little effect on non-target bacteria while disadvantaging target ammonium-oxidising bacteria (which were too rare for statistical analysis). Our results for stream bacteria also contrast with those from soil ecosystems, where DCD application changed neither bacterial diversity nor community composition^26^,^27^. O’Callaghan *et al*.^27^ found that DCD did not affect prevalence of four dominant phyla (Proteobacteria, Firmicutes, Actinobacteria, Acidobacteria), whereas in our experiment Proteobacteria and Firmicutes were disadvantaged by DCD while Actinobacteria and Acidobacteria were favoured. DCD addition also increased evenness at all four taxonomic levels tested, by reducing the prevalence of some abundant taxa, particularly that of Gammaproteobacteria within the orders Pseudomonadales (including *Pseudomonas* and *Actinetobacter*) and Enterobacteriales (including *Erwinia billingiae*), Betaproteobacteria belonging to the Oxalobacteraceae family (including *Massilia varians* and *Janthinobacterium*), and the Firmicutes species *Exiguobacterium sibiricum*. Based on these three studies, DCD may have more wide-ranging effects on bacterial communities in freshwaters compared to soils.

The differences between our findings for DCD and those of the aforementioned studies on soil bacteria may also be related to the length of exposure to DCD. We applied DCD continuously, simulating sustained DCD leaching into streams. By contrast, O’Callaghan *et al*.^27^ and Morales *et al*.^26^ applied DCD once, at the beginning of their experiments, and target bacteria recovered from DCD effects with time. Our continuous DCD application, preventing such a recovery, may have led to the stronger effects observed. Moreover, certain bacteria are known to degrade DCD in soils, especially when other nitrogen sources are limited: a strain of *Mycobacterium* sp^45^., some *Rhodococcus* and *Pseudomonas* bacteria^46^, and *Xanthomonas maltophilia*^47^. It is unknown whether some freshwater bacteria can also degrade DCD, although DCD concentrations declined with time in simulated wetland ponds^29^. It is noteworthy that *Pseudomonas* bacteria showed a negative response to DCD in our experiment, indicating that the more abundant members of this genus in the stream bacterial community were unable to benefit from DCD.

Deposited fine sediment was also a highly pervasive stressor. Sediment addition did not affect bacterial community evenness but increased taxon richness at the phylum and order levels, in contrast with previous findings in the same mesocosm setup. Magbanua *et al*.^42^ and Piggott *et al*.^43^ both found that bacterial taxon richness was unaffected by fine sediment, whereas evenness increased with added sediment in Piggott *et al*.^43^. Sediment addition also changed bacterial community composition (based on the abundant taxa), again supporting our first hypothesis. Cyanobacteria within the order Oscillatoriales (which mainly consisted of a species within the genus *Phormidium*) were positively affected by sediment. Cyanobacteria such as *Phormidium* can be favoured by deposited fine sediment^48^,^49^,^50^, perhaps due to their gliding motility^51^ or their ability to access micronutrients such as iron in the sediment^52^.

Although flow velocity did not influence taxon richness overall, community evenness was increased at the phylum level and many abundant taxa were either positively or negatively affected by the reduction in flow velocity, also supporting our first hypothesis. While our velocity treatments were realistic for small farmland streams^13^, even our “fast” treatment was relatively slow. Therefore, despite the fact that high shear forces from fast flow velocities can affect formation of biofilms^7^,^53^, it is unlikely this was the case in our experiment. More likely, reduced flow velocity affected bacteria through reduced availability of nutrients and oxygen from the water column due to an increase in the substratum boundary layer thickness^32^,^33^,^34^.

Nutrient enrichment was the least influential of the four stressors (in contrast to our first hypothesis). Across all levels of taxonomic resolution, only one abundant order and one common species responded significantly. The order Cytophagales became rarer with added nutrients, while a species of Enterobacteriaceae increased with added nutrients. Cytophagales are found in a wide range of habitats but are also known to adapt to low nutrient concentrations^54^,^55^. The latter could explain why they thrived in our non-enriched treatments compared to enriched ones. The scarcity of nutrient effects in our experiment was surprising because in several previous studies enrichment changed community structure^21^,^22^. Concentrations in our enriched treatments (2923 μg L^−1^ DIN-N, 221 μg L^−1^ SRP-P) were quite high for New Zealand farmland streams^14^. Moreover, they were near the upper end of the concentrations found in a survey of 223 New Zealand streams^22^, where a bacterial community index was positively correlated with logDRP (range: 10–124 μg L^−1^) and negatively with logNOx (47–4268 μg L^−1^). The insensitivity of our bacterial community to nutrient enrichment might be a consequence of studying relative abundances; i.e., while there was little change in community composition there might have been effects on absolute abundances. Alternatively, while nutrient concentrations in our controls were fairly low (124 μg L^−1^ DIN-N, 11 μg L^−1^ SRP-P), they were still higher than in some other streams where nutrient-limitation has been reported^56^,^57^. Therefore, bacteria in controls might not have been limited by the added nutrients and were consequently unaffected by their enrichment.

Interestingly, only one of the stressors affected bacterial taxon richness (fine sediment, discussed above), whereas community evenness increased in response to DCD, fine sediment and flow velocity reduction. Consequently, these stressors may have reduced the competitive advantage of bacterial taxa that dominated under more natural environmental conditions in control mesocosms. These findings contrast with those for many higher plant or animal communities where stress or disturbance often reduces community evenness because they favour highly resistant or resilient species^58^. For bacterial communities, however, similar results were reported from another mesocosm experiment involving the stressors sediment, nutrients and increased temperature^43^. In that experiment, no stressors affected bacterial taxon richness (characterized using ARISA), whereas community evenness increased with sediment addition and with increasing temperature at ambient but not at enriched nutrient levels.

Interactions between stressors occurred at all taxonomic levels (Table S1). The most common interaction was between DCD and flow velocity reduction (1 phylum, 3 orders, 3 genera, 2 abundant species, 4 common species). Taxa showed an antagonistic response^1^, either decreasing with both DCD and reduced velocity (with the combined effect less negative than predicted additively, for example for *Erwinia billingiae* and the associated genus and order), or increasing more strongly with DCD at fast than at reduced velocity (all abundant taxa within the order Verrucomicrobiales). With reduced flow velocity, thickness of the substratum boundary layer increases and nutrient and oxygen availability to the biofilm decreases^32^,^33^, possibly causing these interactive patterns.

DCD also interacted with fine sediment (1 phylum, 2 orders, 1 genus, 1 abundant species, 4 common species). Again, affected taxa showed an antagonistic response, either decreasing with both DCD and sediment (combined effect less negative than predicted additively), or responding more positively to DCD without added sediment. DCD may affect aquatic bacteria directly or indirectly, i.e. through nitrogen enrichment effects of DCD decay products^29^. However, we found very few DCD by nutrient enrichment interactions (2 common species) and DCD effects were much stronger and more common than nutrient effects, suggesting that DCD influenced bacterial communities directly rather than indirectly.

Antagonisms dominated stressor interactions in our experiment. At the community level and at higher taxonomic levels, the dominance of antagonistic interactions may indicate positive co-tolerance of bacterial taxa to the component stressors^59^. According to this concept, taxa remaining in the community at the respective taxonomic level show higher tolerance to additional stressors, thus reducing the effects of additional stressors on community metrics and resulting in antagonistic stressor interactions. Although not specifically proposed by Vinebrooke *et al*.^59^, their concept might be expanded to include intra-specific diversity to provide a potential mechanism of antagonistic interactions revealed at the species level in our experiment.

### Taxonomic resolution versus community coverage

Despite the advances in molecular methods such as high-throughput sequencing, many studies only scratch the surface of the information gained using such techniques. These techniques produce large datasets of sequences with high taxonomic resolution of microbial communities but, out of practicality, often only high taxonomic levels (e.g. phyla) are assessed. While broad generalisations of their ecology can be and often are made at high taxonomic levels, closely related taxa within these higher levels can respond in different ways, with vastly different ecological requirements and tolerances (our study and^60^,^61^).

Our study addresses the open question which level of taxonomic resolution provides the best trade-off between maximising the ability to detect multiple-stressor effects versus the goal of studying the entire bacterial community. We had predicted that genus or species should respond more sensitively than phylum or order because these higher taxonomic levels are more likely to include taxa with opposing responses to stressors, thus diluting or obscuring the overall response. For both single-stressor effects and interactions, this hypothesis was largely rejected. Pervasiveness of single-stressor effects and their effect sizes were both fairly consistent across taxonomic levels (Table 1), while the largest number of interactive effects on abundant taxa was detected for orders (29%, compared to 25% of phyla, 20% of genera and 19% of species). For abundant and common species combined, interactions were detected similarly frequently (30%) as for abundant taxa at the order level.

When evaluating this counter-intuitive result, it is important to keep in mind that amplicon sequencing provides massive datasets with numerous taxa belonging to lower taxonomic levels (e.g. 1,385 genera and 229,192 species in our study). The vast majority of these, including ammonium-oxidising bacteria targeted by DCD, were found in so few mesocosms that their distributions were dominated by zero values (i.e. absences). Consequently, abundance patterns of only a fraction of all genera or species could be analysed statistically. We analysed individual responses of all abundant taxa contributing at least 1% of all sequences (following the definition in Baltar *et al*.^44^), and coverage of the bacterial community decreased strongly with taxonomic resolution. Eight phyla (accounting for 96% of the total number of sequences), 21 orders (84%), 25 genera (64%) and 16 species (28%) were considered as abundant. Even when we included all common species contributing at least 0.3% of all sequences (resulting in a total of 43 species, close to the maximum number we could run our MANOVAs on, and accounting for 41% of all reads), there was still a marked decrease in community coverage from phyla to species. This resulted in considerable loss of information (because distribution patterns of most rare genera or species had to be ignored), and this may explain why lower taxonomic levels did not outperform higher ones when detecting multiple-stressor effects. Low community coverage is most likely a key limitation of all species-level studies of aquatic or terrestrial bacteria. In studies where bacterial species or genera are assessed, percentages of community coverage are rarely given and, where provided, are typically low. For example, when studying a bacterial community impacted by heavy metal pollution using pyrosequencing 16S analysis, Gołębiewski *et al*.^62^ reported that the common genera comprised only 43% of all reads.

For several phyla, the direction of response to the stressors was largely consistent within the phylum, with lower taxonomic groups mostly responding in the same direction (Fig. 4). However, for other phyla including the highly diverse Proteobacteria, responses of orders varied in direction and might therefore be overlooked if conclusions were drawn at the phylum level. In these cases, response direction was often consistent for the genera and species within the different orders. For example, while the abundant orders within the Proteobacteria phylum showed contrasting responses to DCD, all abundant genera and species within the orders of Pseudomonadales and Sphingomonadales responded negatively or positively to DCD, paralleling the respective response pattern at the order level.

When combined, our findings demonstrate that complex responses of genera and species may sometimes be hidden when grouped at phylum level, whereas responses at the order level were generally representative of the responses of the corresponding genera and species. Paralleling our findings at the phylum level, in a recent survey of 12 sites along a Spanish river above and below a reservoir^61^, the distribution patterns of six abundant bacterial classes largely failed to capture the dynamics of their constituent species. Consequently, we conclude that the order level may represent the best compromise between sensitivity/taxonomic resolution and coverage of the bacterial community. Further controlled multiple-stressor experiments on stream bacterial communities are required to determine the extent to which this conclusion can be generalized.

## Methods

### Experimental design

The study was conducted in austral autumn (3 April to 27 May 2013) in an outdoor stream mesocosm system (*ExStream System*^43^) comprising 64 units supplied with water and stream biota from the nearby Kauru River (170°45.9′E, 45°6.4′S, 98 m a.s.l). The Kauru, a 5^th^-order river in the Otago province of New Zealand, drains a 124 km^2^-catchment dominated by tussock grassland and exotic pasture with low-intensity sheep/beef farming. Water was continuously pumped into four header tanks, each of which gravity-fed 16 circular mesocosms (outer diameter 24.5 cm, inner diameter 5.1 cm, volume 3 L, area 450 cm^2^) with 2 L min^−1^ each, allowing continuous immigration and emigration of drifting microbes, periphyton and invertebrates^41^.

To each mesocosm, we added 0.5 L of coarse gravel (*b*-axis 9.2 ± 3.2 mm [SD], resulting in 20–30 mm depth) collected from the river floodplain. Two unglazed terracotta tiles (50 × 50 × 13 mm) placed on this substratum provided a standardised surface for biofilm colonisation. The experiment comprised 27 days with established flow velocity treatments to allow biofilm colonisation followed by 27 days with additional nutrient, sediment and DCD treatments (see below). DCD (two treatment levels) was manipulated at the header tank level, whereas nutrients, flow velocity and sediment (two levels each) were manipulated at the mesocosm level. DCD treatments were randomized within two spatial blocks consisting of two header tanks and 32 mesocosms each. Within DCD treatments, nutrient, flow and sediment treatments were also randomized, resulting in a balanced full-factorial design with four replicates per treatment combination.

Flow velocity treatments were established on day 1 of the experiment and recalibrated daily. Velocities achieved were 0.131 ± 0.003 m s^−1^ (“fast”) and 0.015 ± 0.001 m s^−1^ (“slow”) (means ± SE, measured on days 12 and 25; Flo-Mate, model 2000, Marsh-McBirney Inc., Frederick, Maryland, USA), equivalent to run or pool habitats in small farmland streams^63^. Treatments were established by using a smaller inflow-jet diameter for fast treatments (4.2 *vs*. 10.0 mm inner diameter) while keeping discharge the same for both treatments. This avoided confounding effects on DCD or nutrient concentrations and on un-manipulated physicochemical (e.g. water temperature, dissolved oxygen) and biological variables (e.g. drift of microbes and invertebrates).

On day 28, sediment, nutrient and DCD treatments were established. For sediment treatments, flow was interrupted briefly and 500 g of inorganic, dry river sediment (mean particle size 0.2 mm^63^) added to 32 mesocosms. Fine sediment depth (average of four measurements per mesocosm) and sediment cover (visual estimate) were determined on day 46. Sediment addition resulted in a 7.1 ± 0.2 mm (SE) thick layer of fine sediment covering 93.2 ± 0.5% of the substratum (n = 32).

For nutrient treatments, concentrated solutions of NaNO_3_ and KH_2_PO_4_ were applied to 32 mesocosms at a constant rate of 2 L hour^−1^ with pressure-compensating drippers (RXLD2SC, RX Plastics, Ashburton, NZ). This resulted in concentrations of 2923.4 ± 91.1 μg L^−1^ DIN-N and 220.6 ± 7.0 μg L^−1^ SRP-P (means ± SE, measured on days 28, 41, 52) in nutrient enriched mesocosms, reflecting high but realistic nutrient levels for agricultural streams^14^. Mesocosms with ambient nutrient levels had average concentrations of 123.6 ± 11.5 μg L^−1^ DIN-N and 11.0 ± 1.3 μg L^−1^ SRP-P.

DCD treatments included a control (where concentrations were zero) and a constant concentration of 1.38 ± 0.06 mg L^−1^ (means ± SE; measured on days 29, 31, 35, 37, 41, 53), similar to concentrations found in 1^st^-order streams on a New Zealand dairy farm (R. Storey, NIWA, unpublished data). DCD was dissolved in 140 L tanks and pumped into header tanks using daily-recalibrated piston pumps (FMI CERAMPUMP^®^ Model QBG, Fluid Metering Inc., Syosset, NY, USA).

To enhance colonisation by stream invertebrates underrepresented in the drift^41^, mesocosms were seeded on day 24 with invertebrates from the adjacent river. Invertebrates were collected with standardized kick-net samples from fast (centre) and slow flowing (margins) areas of the same riffles for the respective flow treatments.

### Biofilm sampling

On days 54 and 55 (one block per day), two tiles per mesocosm were collected, individually put on ice, transported to the laboratory and stored in the dark at −20 °C until processing. Following thawing, all biofilm from the top surface of the tiles from each mesocosm was scraped into a container using a toothbrush, rinsed with milli-Q water, and any associated invertebrates removed. Each sample was homogenised using a blender (Omni Mixer, Ivan Sorval Inc., Newton, CT, USA) for one minute, volume-adjusted to 40 ml, and one subsample (4.5 ml) taken for DNA extraction.

### Molecular analyses and sequencing

DNA extraction and amplification of the V3 and V4 hypervariable regions of the 16S rRNA gene are described in the Supporting Information. The resulting samples were de-multiplexed using bcl2fastq script from Illumina sequencer. Raw sequences were paired and quality filtered using QIIME (version 1.7.0) default parameters^64^. Sequences were clustered into species represented by Operational Taxonomic Units (OTUs with 97% sequence similarity) using de novo clustering and the Greengenes database to assign taxonomy^65^. Samples were rarefied (by randomly resampling sequences) to an even depth of 128,600 sequences per sample (minimum sequencing depth). Taxon richness and Simpson’s evenness were calculated at four taxonomic levels: phylum, order, genus and species. For each level, taxa were identified as “abundant” following the definition in Baltar *et al*.^44^ if they contributed at least 1% of the total number of sequences (excluding unassigned taxa). Eight phyla (accounting for 96.2% of all sequences), 21 orders (84.4%), 25 genera (63.5%) and 16 species (28.0%) were considered as abundant and included in the core statistical analyses (see below). To maximise community coverage at the species level, 27 “common” species (after Baltar *et al*.^44^) that contributed 0.3–1.0% of the total number of sequences were also analysed statistically, with the resulting 43 abundant or common species accounting for 40.8% of all sequences. These 43 species were close to the maximum number of related response variables (48) that could be analysed using a MANOVA on our data set given the total sample size of n = 64 and the d.f.s required to model all predictor terms. Potential ammonium oxidising bacteria (targeted by DCD) were also identified but were too rare ( <0.03% of all sequences) to examine statistically. All sequencing data have been uploaded to the NCBI BioSample database (http://www.ncbi.nlm.nih.gov/biosample/) under accession numbers: SAMN05271300-SAMN05271365.

### Statistical analysis

Exploratory Bray-Curtis analyses were performed to examine the four individual stressor effects (DCD, sediment, flow velocity and nutrient enrichment) on the entire bacterial community at each of four taxonomic levels (phylum, order, genus and species) and visualised using NMDS plots. No clear separation of the samples relating to any stressor treatment level was observed, probably due to the complexity of the study design, therefore these results are not presented. Multi-factor ANOVAs were used to quantitatively assess stressor effects on taxon richness and evenness at each taxonomic level. The ANOVA model included the four factors, their interactions and a random block factor (accounting for the two spatial blocks) (see Bruder *et al*.^35^ for details). Equivalent MANOVAs assessed stressor effects on community composition and relative abundances of individual abundant taxa at each taxonomic level and on community composition and relative abundances of abundant plus common taxa at the species level. After exploratory analysis, response variables were transformed as needed to meet assumptions of parametric tests.

All results reported in the text were significant (α = 0.05). Standardized effect sizes (partial eta squared values, range 0–1^66^) are presented for all results with *P* < 0.10 to allow evaluating their biological relevance^67^. No four-way interactions between stressors were significant and are therefore not reported. Block factor results are also not presented because they merely represent background variation unrelated to our research objectives. Data were analysed using R version 3.1.3^68^.

## Additional Information

**How to cite this article:** Salis, R. K. *et al*. High-throughput amplicon sequencing and stream benthic bacteria: identifying the best taxonomic level for multiple-stressor research. *Sci. Rep.* **7**, 44657; doi: 10.1038/srep44657 (2017).

**Publisher's note:** Springer Nature remains neutral with regard to jurisdictional claims in published maps and institutional affiliations.

## Supplementary Material

Supplementary Information

## Figures and Tables

**Table 1:** Frequencies (in %) of significant effects and mean effect sizes (in parentheses) of the four stressors (DCD, sediment addition, flow velocity reduction and nutrient enrichment) and their significant two- or three-way interactions on the abundant (>1.0%) bacterial taxa at the four taxonomic levels and the abundant plus common (>0.3%) bacterial species.

	Phylum	Order	Genus	Species	
	>1.0% *(8 phyla)*	>1.0% *(21 orders)*	>1.0% *(25 genera)*	>1.0% *(16 species)*	>0.3% *(43 species)*
DCD	75% (0.34)	76% (0.34)	76% (0.32)	88% (0.27)	88% (0.27)
Sediment	75% (0.22)	43% (0.16)	48% (0.17)	50% (0.16)	60% (0.20)
Flow velocity	25% (0.16)	43% (0.17)	36% (0.16)	19% (0.28)	30% (0.24)
Nutrients	0%	5% (0.20)	0%	0%	2% (0.37)
DCD × Sed	13% (0.11)	10% (0.12)	4% (0.10)	6% (0.10)	12% (0.11)
DCD × Flow	13% (0.09)	14% (0.12)	12% (0.11)	13% (0.17)	14% (0.14)
DCD × Nut	0%	0%	0%	0%	5% (0.10)
Sed × Flow	0%	5% (0.14)	0%	0%	2% (0.08)
Sed × Nut	0%	0%	4% (0.12)	0%	2% (0.19)
Nut × Flow	0%	5% (0.13)	0%	0%	0%
DCD × Sed × Nut	0%	0%	0%	0%	2% (0.09)

No four-way interactions were significant.
