# Supplementary material for: High-throughput amplicon sequencing and stream benthic bacteria: identifying the best taxonomic level for multiple-stressor research
Source: Sci Rep. 2017 Mar 22;7:44657. doi: 10.1038/srep44657 (PMC5361126; doi:10.1038/srep44657)
Supplement: Supplementary Information [file srep44657-s1.pdf]

# High-throughput amplicon sequencing and stream benthic bacteria: identifying the best taxonomic level for multiple-stressor research

R.K. Salis[1*], A. Bruder[1,3], J.J. Piggott[1,4], T. C. Summerfield[2] & C.D. Matthaei[1]

[1] Department of Zoology, University of Otago, Dunedin, New Zealand

[2] Department of Botany, University of Otago, Dunedin, New Zealand

[3] Present address: Institute of Earth Sciences, University of Applied Sciences and Arts of Southern Switzerland, Canobbio, Switzerland

[4] School of Natural Sciences, Trinity College Dublin, the University of Dublin, Dublin 2, Ireland

* corresponding author

## Supplementary Information

This document contains information on DNA extraction and amplification procedures, descriptions of all observed significant interactions for bacterial response variables with corresponding figures, and full taxonomic information on the common phyla, orders, genera and species (OTU with clustering at 97% similarity).

### 1. DNA extraction and amplification

DNA was extracted from the 64 samples using a bead beater extraction protocol (Miller et al., 1999). Amplification of the V3 and V4 hypervariable regions of the 16S rRNA gene was performed using oligonucleotide primers (Klindworth et al., 2013) modified by the addition of Illumina Tags (underlined): S-D-Bact-0341-b-S-17 (5′-ACGACGCTCTTCCGATCTCCTAC GGGNGGCWGCAG-3′) and S-D-Bact-0785-a-A-21 (5′-CGTGTGCTCTTCCGATCTGACT ACHVGGGTATCTA-3′), resulting in amplicons that contained a 16S rRNA gene region of ~440 bp. The HiFi HotStart PCR kit (KAPA Biosystems, Boston, MA, USA) was used with the following protocol: 0.2 mM dNTPs, 40 ng of DNA template, 0.02 U/µl KAPA HiFi Taq, 0.2 µM of each primer in a total volume of 50 µl. PCR conditions were: initial denaturation at 95°C for 5 min; followed by 27 cycles of 95°C for 1 min, 56°C (1 min) and 72°C (1 min); final extension at 72°C (5 min). Amplified products were purified using Agencourt AMPure XP purification kit (protocol 000387v001; Beckman Coulter, Brea, CA, USA) and quantified using Qubit dsDNA High Sensitivity Assay (Agilent Technologies, Palo Alto, CA, USA). Then a second round of PCR was performed using TruSeq® RNA PCR Primers (RPI1-33; Illumina, San Diego, CA, USA) to attach 6-bp multiplexing indices and Illumina sequencing adapters using the following protocol: 0.2 mM dNTPs, 2 ng of the purified PCR product, 0.02 U/µl KAPA HiFi Taq, 0.2 µM of each primer in a total volume of 50 µl. PCR conditions were: initial denaturation at 95°C for 20 sec; 10 cycles of 95°C (20 sec), 55°C (20 sec) and 68°C (20 sec); final extension at 68°C (40 sec). Amplicons were

purified and quantified as above and run on the MiSeq Illumina sequencing platform, producing 2 × 250 bp reads (Illumina, San Diego, CA, USA).

*References*

Klindworth A, Pruesse E, Schweer T, Peplies J, Quast C, Horn M et al. (2013). Evaluation of general 16S ribosomal RNA gene PCR primers for classical and next-generation sequencing-based diversity studies. Nucleic Acids Research 41: e1 doi:10.1093/nar/gks1808.

Miller D, Bryant J, Madsen E & Ghiorse W (1999). Evaluation and optimization of DNA extraction and purification procedures for soil and sediment samples. Applied and Environmental Microbiology 65: 4715-4724.

## 2. Taxonomic classifications of the abundant and common phyla, orders, genera and species

**Abundant Phyla (8)**

| Phylum | % of all sequences |
|---|---|
| Proteobacteria | 57.5 |
| Bacteroidetes | 16.0 |
| Verrucomicrobia | 6.3 |
| Planctomycetes | 5.2 |
| Actinobacteria | 3.4 |
| Cyanobacteria | 3.1 |
| Acidobacteria | 2.4 |
| Firmicutes | 2.2 |
| **Total** | **96.2** |

**Abundant Orders (21)**

| Phylum | Class | Order | % of all sequences |
|---|---|---|---|
| Proteobacteria | Alphaproteobacteria | Sphingomonadales | 8.0 |
| Proteobacteria | Alphaproteobacteria | Rhodobacterales | 4.7 |
| Proteobacteria | Alphaproteobacteria | Rhizobiales | 3.9 |
| Proteobacteria | Alphaproteobacteria | Caulobacterales | 1.8 |
| Proteobacteria | Betaproteobacteria | Burkholderiales | 12.6 |
| Proteobacteria | Betaproteobacteria | SC-I-84 | 1.8 |
| Proteobacteria | Gammaproteobacteria | Pseudomonadales | 12.4 |
| Proteobacteria | Gammaproteobacteria | Enterobacteriales | 3.6 |
| Proteobacteria | Gammaproteobacteria | Xanthomonadales | 2.8 |
| Proteobacteria | Gammaproteobacteria | Legionellales | 1.4 |
| Bacteroidetes | [Saprospirae] | [Saprospirales] | 4.8 |
| Bacteroidetes | Cytophagia | Cytophagales | 2.0 |
| Bacteroidetes | Flavobacteriia | Flavobacteriales | 6.7 |
| Bacteroidetes | Sphingobacteriia | Sphingobacteriales | 2.3 |
| Verrucomicrobia | [Spartobacteria] | [Chthoniobacterales] | 1.4 |
| Verrucomicrobia | Verrucomicrobiae | Verrucomicrobiales | 4.6 |
| Planctomycetes | Phycisphaerae | WD2101 | 1.0 |
| Planctomycetes | Planctomycetia | Pirellulales | 2.4 |
| Actinobacteria | Actinobacteria | Actinomycetales | 2.2 |
| Cyanobacteria | Oscillatoriophycideae | Oscillatoriales | 1.8 |
| Firmicutes | Bacilli | Bacillales | 2.1 |
| | | **Total** | **84.4** |

**Abundant Genera (25)**

| Phylum | Class | Order | Family | Genus | % of all sequences |
|---|---|---|---|---|---|
| Proteobacteria | Alphaproteobacteria | Caulobacterales | Caulobacteraceae | UA | 1.3 |
| Proteobacteria | Alphaproteobacteria | Rhizobiales | UA | UA | 1.4 |
| Proteobacteria | Alphaproteobacteria | Rhodobacterales | Rhodobacteraceae | *Rhodobacter* | 4.2 |
| Proteobacteria | Alphaproteobacteria | Sphingomonadales | Sphingomonadaceae | *Novosphingobium* | 1.7 |
| Proteobacteria | Alphaproteobacteria | Sphingomonadales | Sphingomonadaceae | *Sphingomonas* | 1.2 |
| Proteobacteria | Alphaproteobacteria | Sphingomonadales | UA | UA | 1.5 |
| Proteobacteria | Betaproteobacteria | Burkholderiales | Comamonadaceae | UA | 2.8 |
| Proteobacteria | Betaproteobacteria | Burkholderiales | Oxalobacteraceae | *Massilia* | 4.7 |
| Proteobacteria | Betaproteobacteria | Burkholderiales | Oxalobacteraceae | *Janthinobacterium* | 3.5 |
| Proteobacteria | Betaproteobacteria | SC-I-84 | UA | UA | 1.8 |
| Proteobacteria | Gammaproteobacteria | Enterobacteriales | Enterobacteriaceae | UA | 1.5 |
| Proteobacteria | Gammaproteobacteria | Enterobacteriales | Enterobacteriaceae | *Erwinia* | 1.5 |
| Proteobacteria | Gammaproteobacteria | Pseudomonadales | Moraxellaceae | *Acinetobacter* | 3.7 |
| Proteobacteria | Gammaproteobacteria | Pseudomonadales | Pseudomonadaceae | *Pseudomonas* | 6.9 |
| Proteobacteria | Gammaproteobacteria | Pseudomonadales | Pseudomonadaceae | UA | 1.7 |
| Proteobacteria | Gammaproteobacteria | Xanthomonadales | Xanthomonadaceae | UA | 1.7 |
| Bacteroidetes | Flavobacteriia | Flavobacteriales | Flavobacteriaceae | UA | 3.2 |
| Bacteroidetes | Flavobacteriia | Flavobacteriales | Flavobacteriaceae | *Flavobacterium* | 2.8 |
| Bacteroidetes | Sphingobacteriia | Sphingobacteriales | Chitinophagaceae | UA | 4.1 |
| Bacteroidetes | Sphingobacteriia | Sphingobacteriales | Sphingobacteriaceae | *Pedobacter* | 1.8 |
| Verrucomicrobia | Verrucomicrobiae | Verrucomicrobiales | Verrucomicrobiaceae | *Luteolibacter* | 3.3 |
| Verrucomicrobia | Verrucomicrobiae | Verrucomicrobiales | Verrucomicrobiaceae | UA | 1.3 |
| Planctomycetes | Planctomycetia | Pirellulales | Pirellulaceae | UA | 2.2 |
| Cyanobacteria | Oscillatoriophycideae | Oscillatoriales | Phormidiaceae | *Phormidium* | 1.8 |
| Firmicutes | Bacilli | Bacillales | [Exiguobacteraceae] | *Exiguobacterium* | 1.9 |
| | | | | **Total** | **63.5** |

| **Abundant Species (16)** | | | | | | **% of all sequences** |
|---|---|---|---|---|---|---|
| **Phylum** | **Class** | **Order** | **Family** | **Genus** | **Species** | |
| Proteobacteria | Alphaproteobacteria | Rhodobacterales | Rhodobacteraceae | *Rhodobacter* | UA | 2 |
| Proteobacteria | Alphaproteobacteria | Sphingomonadales | Sphingomonadaceae | *Novosphingobium* | UA | 1.3 |
| Proteobacteria | Alphaproteobacteria | Sphingomonadales | Sphingomonadaceae | *Sphingopyxis* | UA | 1 |
| Proteobacteria | Betaproteobacteria | Burkholderiales | Comamonadaceae | UA | UA | 1.1 |
| Proteobacteria | Betaproteobacteria | Burkholderiales | Oxalobacteraceae | *Janthinobacterium* | *lividum* | 1.3 |
| Proteobacteria | Betaproteobacteria | Burkholderiales | Oxalobacteraceae | *Massilia* | *varians* | 2.2 |
| Proteobacteria | Gammaproteobacteria | Enterobacteriales | Enterobacteriaceae | *Erwinia* | *billingiae* | 1.3 |
| Proteobacteria | Gammaproteobacteria | Pseudomonadales | Moraxellaceae | *Acinetobacter* | UA | 1.4 |
| Proteobacteria | Gammaproteobacteria | Pseudomonadales | Pseudomonadaceae | *Pseudomonas* | UA 1 | 3 |
| Proteobacteria | Gammaproteobacteria | Pseudomonadales | Pseudomonadaceae | *Pseudomonas* | UA 2 | 1.8 |
| Proteobacteria | Gammaproteobacteria | Pseudomonadales | Pseudomonadaceae | *Pseudomonas* | UA 3 | 1.5 |
| Bacteroidetes | Flavobacteriia | Flavobacteriales | Flavobacteriaceae | *Flavobacterium* | UA | 3.4 |
| Bacteroidetes | Sphingobacteriia | Sphingobacteriales | Sphingobacteriaceae | *Pedobacter* | UA | 1.3 |
| Verrucomicrobia | Verrucomicrobiae | Verrucomicrobiales | Verrucomicrobiaceae | *Luteolibacter* | UA | 1.8 |
| Cyanobacteria | Oscillatoriophycideae | Oscillatoriales | Phormidiaceae | *Phormidium* | UA | 1.7 |
| Firmicutes | Bacilli | Bacillales | Exiguobacteraceae | *Exiguobacterium* | *sibiricum* | 1.8 |
| | | | | | **Total** | **28.0** |
| **Common Species (27)** | | | | | | |
| Actinobacteria | Actinobacteria | Actinomycetales | Micrococcaceae | *UA* | UA | 0.9 |
| Proteobacteria | Betaproteobacteria | Burkholderiales | Oxalobacteraceae | *Janthinobacterium* | UA | 0.9 |
| Proteobacteria | Gammaproteobacteria | Enterobacteriales | Enterobacteriaceae | UA | UA | 0.8 |
| Proteobacteria | Alphaproteobacteria | Sphingobacteriales | Sphingomonadaceae | | UA | 0.6 |
| Proteobacteria | Gammaproteobacteria | Xanthomonadales | Xanthomonadaceae | UA | UA | 0.6 |
| Proteobacteria | Alphaproteobacteria | Caulobacterales | Caulobacteraceae | UA | UA | 0.6 |
| Proteobacteria | Gammaproteobacteria | Pseudomonadales | Moraxellaceae | *Acinetobacter* | UA | 0.5 |
| Proteobacteria | Alphaproteobacteria | Rhodobacterales | Rhodobacteraceae | *Rhodobacter* | UA | 0.5 |
| Proteobacteria | Alphaproteobacteria | Sphingobacteriales | Sphingomonadaceae | *Kaistobacter* | UA | 0.5 |
| Proteobacteria | Alphaproteobacteria | Sphingobacteriales | Sphingomonadaceae | *Sphingomonas* | *echinoides* | 0.5 |
| Proteobacteria | Betaproteobacteria | Betaproteobacteria | SC-I-84 | UA | UA | 0.5 |
| Proteobacteria | Betaproteobacteria | Burkholderiales | Oxalobacteraceae | UA | UA | 0.5 |
| Proteobacteria | Gammaproteobacteria | Pseudomonadales | Pseudomonadaceae | *Pseudomonas* | UA | 0.5 |
| Bacteroidetes | Saprospirae | Saprospirales | Chitinophagaceae | UA | UA | 0.4 |
| Bacteroidetes | Flavobacteriia | Flavobacteriales | Flavobacteriaceae | *Flavobacterium* | UA | 0.4 |
| Planctomycetes | Planctomycetia | Pirellulales | Pirellulaceae | UA | UA | 0.4 |
| Proteobacteria | Alphaproteobacteria | Caulobacterales | Caulobacteraceae | UA | UA | 0.4 |
| Bacteroidetes | Flavobacteriia | Flavobacteriales | Flavobacteriaceae | *Flavobacterium* | UA | 0.4 |
| Proteobacteria | Alphaproteobacteria | Rhodobacterales | Rhodobacteraceae | *Rhodobacter* | UA | 0.4 |
| Cyanobacteria | Synechococcophycideae | Synechococcales | Synechococcaceae | *Synechococcus* | UA | 0.4 |
| Proteobacteria | Gammaproteobacteria | Enterobacteriales | Enterobacteriaceae | UA | UA | 0.4 |
| Proteobacteria | Alphaproteobacteria | Sphingobacteriales | Sphingomonadaceae | *Sphingomonas* | UA | 0.3 |
| Proteobacteria | Gammaproteobacteria | Pseudomonadales | Moraxellaceae | *Acinetobacter* | UA | 0.3 |
| Verrucomicrobia | Verrucomicrobiae | Verrucomicrobiales | Verrucomicrobiaceae | UA | UA | 0.3 |
| Proteobacteria | Gammaproteobacteria | Enterobacteriales | Enterobacteriaceae | UA | UA | 0.3 |
| Verrucomicrobia | Verrucomicrobiae | Verrucomicrobiales | Verrucomicrobiaceae | *Luteolibacter* | UA | 0.3 |
| Proteobacteria | Betaproteobacteria | Burkholderiales | Oxalobacteraceae | *Janthinobacterium* | UA | 0.3 |
| | | | | **Total** (abundant plus | common) | **40.8** |

**Table S1.** Summary of ANOVA/MANOVAs examining the effects of DCD, fine sediment, flow velocity reduction, nutrients and their interactions (all 2-way interactions plus the only significant 3-way interaction) on bacterial taxon richness, evenness and abundant taxa at the phylum, order, genus and species levels. *P*-values < 0.05 are indicated in bold. Effect sizes (ES) and response directions (dir.) are given for all response variables where *P* < 0.10. UAG = unassigned genus; UAS = unassigned species (See Section 2 above for full taxonomic information).

| | | DCD | | | Sediment | | | Flow | | | Nutrients | | | DCD×Sed | | DCD×Flow | | DCD×Nut | | Sed×Flow | | Sed×Nut | | Nut×Flow | |
|---|---|---|---|---|---|---|---|---|---|---|---|---|---|---|---|---|---|---|---|---|---|---|---|---|---|
| **PHYLUM LEVEL** | | ***P*** | **ES** | **dir.** | ***P*** | **ES** | **dir.** | ***P*** | **ES** | **dir.** | ***P*** | **ES** | **dir.** | ***P*** | **ES** | ***P*** | **ES** | ***P*** | **ES** | ***P*** | **ES** | ***P*** | **ES** | ***P*** | **ES** |
| Taxon richness | | 0.633 | | | **0.018** | 0.11 | + | 0.156 | | | 0.341 | | | 0.320 | | 0.602 | | 0.320 | 0.08 | 0.633 | | 0.299 | | 0.182 | |
| Simpsons evenness | | **<0.001** | 0.39 | + | 0.101 | 0.06 | + | **0.048** | 0.08 | + | 0.741 | | | 0.708 | | 0.990 | | 0.888 | | 0.617 | | **0.049** | 0.08 | 0.905 | |
| *MANOVA* (phyla > 1%, n=8) | | **<0.001** | 0.63 | | **<0.001** | 0.84 | | **<0.001** | 0.63 | | 0.152 | | | **0.008** | 0.38 | **0.023** | 0.34 | 0.130 | | 0.057 | 0.30 | 0.386 | | 0.580 | |
| **Abundant Phyla** | % | | | | | | | | | | | | | | | | | | | | | | | | |
| Proteobacteria | 57.5 | **<0.001** | 0.51 | - | **<0.001** | 0.21 | - | **0.042** | 0.09 | - | 0.800 | | | **0.022** | 0.11 | 0.445 | | 0.583 | | 0.571 | | 0.192 | | 0.482 | |
| Bacteroidetes | 16.0 | 0.067 | 0.07 | + | **0.012** | 0.13 | + | **<0.001** | 0.23 | + | 0.592 | | | 0.954 | | 0.666 | | 0.597 | | 0.971 | | 0.548 | | 0.548 | |
| Verrucomicrobia | 6.3 | **<0.001** | 0.36 | + | 0.847 | | | 0.156 | | | 0.141 | | | 0.411 | | **0.037** | 0.09 | 0.209 | | 0.681 | | 0.858 | | 0.218 | |
| Planctomycetes | 5.2 | **<0.001** | 0.37 | + | **0.001** | 0.20 | - | 0.699 | | | 0.076 | 0.07 | - | 0.143 | | 0.335 | | 0.341 | | 0.218 | | 0.713 | | 0.276 | |
| Actinobacteria | 3.4 | **<0.001** | 0.27 | + | **<0.001** | 0.28 | + | 0.596 | | | 0.982 | | | 0.514 | | 0.737 | | 0.978 | | 0.952 | | 0.709 | | 0.227 | |
| Cyanobacteria | 3.1 | 0.160 | | | 0.190 | | | 0.556 | | | 0.222 | | | 0.661 | | 0.322 | | 0.855 | | 0.179 | | 0.587 | | 0.259 | |
| Acidobacteria | 2.4 | **<0.001** | 0.27 | + | **<0.001** | 0.35 | + | 0.374 | | | 0.149 | | | 0.448 | | 0.951 | | 0.726 | | 0.842 | | 0.263 | | 0.219 | |
| Firmicutes | 2.2 | **<0.001** | 0.24 | - | **0.008** | 0.14 | + | 0.131 | | | 0.059 | 0.07 | + | 0.054 | | 0.135 | | 0.125 | | 0.141 | | 0.257 | | 0.780 | |
| | 96.2% | | | | | | | | | | | | | | | | | | | | | | | | |

| | | DCD | | | Sediment | | | Flow | | | Nutrients | | | DCD×Sed | | DCD×Flow | | DCD×Nut | | Sed×Flow | | Sed×Nut | | Nut×Flow | |
|---|---|---|---|---|---|---|---|---|---|---|---|---|---|---|---|---|---|---|---|---|---|---|---|---|---|
| **ORDER LEVEL** | | ***P*** | **ES** | **dir.** | ***P*** | **ES** | **dir.** | ***P*** | **ES** | **dir.** | ***P*** | **ES** | **dir.** | ***P*** | **ES** | ***P*** | **ES** | ***P*** | **ES** | ***P*** | **ES** | ***P*** | **ES** | ***P*** | **ES** |
| Taxon richness | | 0.532 | | | **0.018** | 0.11 | + | 0.381 | | | 0.250 | | | 0.284 | | 0.436 | | 0.349 | | 0.677 | | 0.339 | | 0.293 | |
| Simpsons evenness | | **<0.001** | 0.57 | + | 0.724 | | | 0.533 | | | 0.702 | | | 0.851 | | 0.519 | | 0.707 | | 0.613 | | 0.770 | | 0.976 | |
| *MANOVA* (orders > 1%, n=21) | | **<0.001** | 0.87 | | **<0.001** | 0.93 | | **<0.001** | 0.84 | | **0.015** | 0.66 | | **0.016** | 0.65 | 0.101 | | **0.013** | 0.66 | **<0.001** | 0.79 | 0.058 | 0.60 | 0.276 | |
| **Abundant Orders** | % | | | | | | | | | | | | | | | | | | | | | | | | |
| Burkholderiales | 12.6 | **<0.001** | 0.38 | - | 0.576 | | | 0.257 | | | 0.228 | | | 0.950 | | 0.198 | | 0.511 | | 0.903 | | 0.429 | | 0.333 | |
| Pseudomonadales | 12.4 | **<0.001** | 0.45 | - | 0.124 | | | 0.928 | | | 0.486 | | | 0.097 | 0.06 | 0.184 | | 0.612 | | 0.873 | | 0.677 | | 0.257 | |
| Sphingomonadales | 8.0 | **<0.001** | 0.35 | + | 0.217 | | | 0.830 | | | 0.148 | | | 0.940 | | 0.524 | | 0.692 | | 0.487 | | 0.216 | | 0.695 | |
| Flavobacteriales | 6.7 | 0.985 | | | 0.072 | 0.07 | + | **0.002** | 0.18 | + | 0.906 | | | 0.877 | | 0.867 | | 0.386 | | 0.693 | | 0.471 | | 0.973 | |
| Saprospirales | 4.8 | **<0.001** | 0.26 | + | **0.019** | 0.11 | + | 0.797 | | | 0.406 | | | 0.929 | | 0.206 | | 0.568 | | 0.645 | | 0.268 | | 0.194 | |
| Rhodobacterales | 4.7 | **0.001** | 0.20 | + | 0.247 | | | 0.075 | 0.07 | - | 0.957 | | | 0.476 | | 0.413 | | 0.869 | | 0.477 | | 0.450 | | 0.633 | |
| Verrucomicrobiales | 4.6 | **<0.001** | 0.36 | + | 0.886 | | | **0.042** | 0.08 | - | 0.245 | | | 0.405 | | **0.019** | 0.11 | 0.150 | | 0.449 | | 0.770 | | 0.168 | |
| Rhizobiales | 3.9 | **<0.001** | 0.42 | + | 0.577 | | | 0.200 | | | 0.521 | | | 0.168 | | 0.684 | | 0.926 | | 0.928 | | 0.886 | | 0.361 | |
| Enterobacteriales | 3.6 | **<0.001** | 0.33 | - | **0.012** | 0.13 | - | **0.006** | 0.15 | - | 0.199 | | | 0.113 | | **0.013** | 0.13 | 0.693 | | 0.533 | | 0.859 | | 0.679 | |
| Xanthomonadales | 2.8 | **<0.001** | 0.39 | + | **0.022** | 0.11 | + | 0.286 | | | 0.889 | | | 0.465 | | 0.206 | | 0.552 | | 0.294 | | 0.396 | | 0.821 | |
| Pirellulales | 2.4 | **<0.001** | 0.42 | + | **<0.001** | 0.39 | - | 0.704 | | | 0.083 | 0.06 | - | **0.035** | 0.09 | 0.322 | | 0.516 | | 0.539 | | 0.501 | | 0.319 | |
| Sphingobacteriales | 2.3 | 0.253 | | | 0.126 | | | **0.042** | 0.08 | + | 0.735 | | | 0.516 | | 0.895 | | 0.911 | | 0.819 | | 0.482 | | 0.592 | |
| Actinomycetales | 2.2 | **0.018** | 0.11 | + | **<0.001** | 0.21 | + | **0.045** | 0.08 | + | 0.988 | | | 0.454 | | 0.265 | | 0.932 | | 0.989 | | 0.614 | | 0.363 | |
| Bacillales | 2.1 | **<0.001** | 0.24 | - | **0.008** | 0.14 | + | 0.151 | | | 0.058 | 0.07 | + | 0.056 | 0.08 | 0.125 | | 0.128 | | 0.134 | | 0.255 | | 0.806 | |
| Cytophagales | 2.0 | 0.340 | | | 0.087 | 0.06 | - | **0.001** | 0.20 | + | **0.001** | 0.20 | - | 0.594 | | 0.480 | | 0.821 | | 0.121 | | 0.162 | | **0.010** | 0.13 |
| Caulobacterales | 1.8 | 0.698 | | | 0.183 | | | **<0.001** | 0.32 | + | 0.876 | | | 0.341 | | 0.651 | | 0.534 | | 0.813 | | 0.793 | | 0.569 | |
| Oscillatoriales | 1.8 | 0.428 | | | **0.005** | 0.16 | + | 0.904 | | | 0.366 | | | 0.755 | | 0.297 | | 0.927 | | 0.279 | | 0.335 | | 0.151 | |
| SC-I-84 (Betaproteobacteria) | 1.8 | **<0.001** | 0.40 | + | **0.048** | 0.08 | + | **0.022** | 0.11 | - | 0.470 | | | 0.343 | | 0.307 | | 0.467 | | 0.301 | | 0.550 | | 0.525 | |
| Legionellales | 1.4 | **<0.001** | 0.48 | + | **0.012** | 0.13 | - | **<0.001** | 0.36 | - | 0.665 | | | **0.005** | 0.16 | **0.010** | 0.13 | 0.914 | | 0.101 | | 0.718 | | 0.562 | |
| Chthoniobacterales | 1.4 | **<0.001** | 0.38 | + | 0.880 | | | 0.788 | | | 0.059 | 0.07 | - | 0.229 | | 0.263 | | 0.441 | | 0.664 | | 0.676 | | 0.642 | |
| WD2101 (Phycisphaerae) | 1.0 | **<0.001** | 0.26 | + | 0.866 | | | 0.214 | | | 0.076 | 0.07 | - | 0.839 | | 0.248 | | 0.071 | 0.07 | **0.008** | 0.14 | 0.505 | | 0.122 | |
| | 84.4% | | | | | | | | | | | | | | | | | | | | | | | | |

| GENUS LEVEL | | DCD *P* | ES | dir. | Sediment *P* | ES | dir. | Flow *P* | ES | dir. | Nutrients *P* | ES | dir. | DCD×Sed *P* | ES | DCD×Flow *P* | ES | DCD×Nut *P* | ES | Sed×Flow *P* | ES | Sed×Nut *P* | ES | Nut×Flow *P* | ES |
|---|---|---|---|---|---|---|---|---|---|---|---|---|---|---|---|---|---|---|---|---|---|---|---|---|---|
| Taxon richness | | 0.591 | | | 0.156 | | | 0.412 | | | 0.413 | | | 0.292 | | 0.384 | | 0.375 | | 0.662 | | 0.358 | | 0.382 | |
| Simpsons evenness | | **<0.001** | 0.53 | + | 0.516 | | | 0.249 | | | 0.965 | | | 0.780 | | 0.371 | | 0.547 | | 0.363 | | 0.223 | | 0.763 | |
| | | | | | | | | | | | | | | | | | | | | | | | | | |
| *MANOVA* (genera > 1%, n=25) | | **<0.001** | 0.89 | | **<0.001** | 0.94 | | **<0.001** | 0.89 | | 0.198 | | | 0.070 | 0.67 | 0.332 | | 0.242 | | **<0.001** | 0.84 | 0.486 | | 0.122 | |
| **Abundant Genera** | % | | | | | | | | | | | | | | | | | | | | | | | | |
| *Pseudomonas* | 6.9 | **<0.001** | 0.33 | - | 0.810 | | | 0.540 | | | 0.215 | | | 0.407 | | 0.331 | | 0.215 | | 0.786 | | 0.304 | | 0.489 | |
| *Massilia* | 4.7 | **<0.001** | 0.31 | - | **0.012** | 0.13 | - | **0.027** | 0.10 | + | 0.215 | | | 0.511 | | 0.309 | | 0.179 | | 0.989 | | 0.612 | | 0.158 | |
| *Rhodobacter* | 4.2 | **0.001** | 0.20 | + | 0.176 | | | **0.047** | 0.08 | - | 0.850 | | | 0.392 | | 0.384 | | 0.818 | | 0.433 | | 0.483 | | 0.586 | |
| Chitinophagaceae UAG | 4.1 | **<0.001** | 0.25 | + | **0.005** | 0.16 | + | 0.622 | | | 0.370 | | | 0.779 | | 0.209 | | 0.539 | | 0.582 | | 0.241 | | 0.181 | |
| *Acinetobacter* | 3.7 | **<0.001** | 0.29 | - | **0.006** | 0.15 | - | 0.733 | | | 0.755 | | | 0.057 | 0.07 | 0.227 | | 0.530 | | 0.516 | | 0.690 | | 0.231 | |
| *Janthinobacterium* | 3.5 | **<0.001** | 0.40 | - | 0.351 | | | 0.102 | | | 0.961 | | | 0.524 | | 0.903 | | 0.984 | | 0.572 | | 0.611 | | 0.544 | |
| *Luteolibacter* | 3.3 | **<0.001** | 0.31 | + | 0.568 | | | 0.162 | | | 0.136 | | | 0.487 | | **0.016** | 0.12 | 0.156 | | 0.337 | | 0.653 | | 0.073 | 0.07 |
| Flavobacteriaceae UAG | 3.2 | 0.057 | 0.08 | + | 0.630 | | | 0.334 | | | 0.682 | | | 0.824 | | 0.507 | | 0.547 | | 0.975 | | 0.413 | | 0.686 | |
| *Flavobacterium* | 2.8 | **0.001** | 0.20 | - | **0.004** | 0.16 | + | **<0.001** | 0.27 | + | 0.393 | | | 0.327 | | 0.292 | | 0.627 | | 0.299 | | 0.773 | | 0.376 | |
| Comamonadaceae UAG | 2.8 | 0.177 | | | **0.016** | 0.12 | + | **<0.001** | 0.30 | - | 0.223 | | | 0.841 | | 0.109 | | 0.449 | | 0.551 | | 0.593 | | 0.226 | |
| Pirellulaceae UAG | 2.2 | **<0.001** | 0.42 | + | **<0.001** | 0.42 | - | 0.706 | | | 0.073 | 0.07 | - | **0.028** | 0.10 | 0.341 | | 0.528 | | 0.624 | | 0.487 | | 0.313 | |
| *Exiguobacterium* | 1.9 | **<0.001** | 0.23 | - | **0.011** | 0.13 | + | 0.128 | | | 0.068 | 0.07 | + | 0.064 | 0.07 | 0.109 | | 0.140 | | 0.139 | | 0.291 | | 0.827 | |
| *Phormidium* | 1.8 | 0.431 | | | **0.005** | 0.16 | + | 0.903 | | | 0.365 | | | 0.756 | | 0.297 | | 0.927 | | 0.278 | | 0.335 | | 0.151 | |
| *Pedobacter* | 1.8 | 0.303 | | | 0.275 | | | 0.093 | 0.06 | + | 0.662 | | | 0.493 | | 0.958 | | 0.829 | | 0.959 | | 0.439 | | 0.568 | |
| SC-I-84 UAG | 1.8 | **<0.001** | 0.40 | + | **0.048** | 0.08 | + | **0.022** | 0.11 | - | 0.470 | | | 0.343 | | 0.307 | | 0.467 | | 0.301 | | 0.550 | | 0.525 | |
| Xanthomonadaceae UAG | 1.7 | **<0.001** | 0.38 | + | 0.060 | 0.07 | + | 0.158 | | | 0.716 | | | 0.923 | | 0.091 | 0.06 | 0.468 | | 0.132 | | 0.560 | | 0.870 | |
| *Novosphingobium* | 1.7 | 0.064 | 0.07 | + | **<0.001** | 0.31 | + | 0.736 | | | 0.065 | 0.07 | - | 0.092 | 0.06 | 0.828 | | 0.457 | | 0.666 | | **0.014** | 0.12 | 0.980 | |
| Pseudomonadaceae UAG | 1.7 | **<0.001** | 0.44 | - | 0.544 | | | 0.784 | | | 0.309 | | | 0.391 | | 0.416 | | 0.329 | | 0.762 | | 0.671 | | 0.496 | |
| Sphingomonadales UAG | 1.5 | **<0.001** | 0.36 | + | 0.835 | | | 0.800 | | | 0.389 | | | 0.689 | | 0.373 | | 0.982 | | 0.636 | | 0.118 | | 0.946 | |
| Enterobacteriaceae UAG | 1.5 | **<0.001** | 0.34 | - | **0.024** | 0.10 | - | 0.058 | 0.07 | - | 0.083 | 0.06 | + | 0.175 | | 0.052 | 0.08 | 0.502 | | 0.969 | | 0.612 | | 0.539 | |
| *Erwinia* | 1.5 | **<0.001** | 0.28 | - | **0.014** | 0.12 | - | **0.002** | 0.18 | - | 0.391 | | | 0.117 | | **0.011** | 0.13 | 0.939 | | 0.319 | | 0.985 | | 0.831 | |
| Rhizobiales UAG | 1.4 | **<0.001** | 0.34 | + | 0.239 | | | **0.039** | 0.09 | - | 0.809 | | | 0.292 | | 0.275 | | 0.400 | | 0.355 | | 0.651 | | 0.912 | |
| Caulobacteraceae UAG | 1.3 | 0.713 | | | 0.134 | | | **0.005** | 0.16 | + | 0.899 | | | 0.246 | | 0.390 | | 0.757 | | 0.996 | | 0.836 | | 0.447 | |
| Verrucomicrobiaceae UAG | 1.3 | **<0.001** | 0.40 | + | 0.065 | 0.07 | - | **0.002** | 0.19 | - | 0.874 | | | 0.290 | | **0.050** | 0.08 | 0.184 | | 0.818 | | 0.984 | | 0.815 | |
| *Sphingomonas* | 1.2 | **0.003** | 0.17 | + | 0.218 | | | 0.693 | | | 0.089 | 0.06 | - | 0.097 | 0.06 | 0.465 | | 0.267 | | 0.119 | | 0.332 | | 0.137 | |
| | 63.5% | | | | | | | | | | | | | | | | | | | | | | | | |

| | | DCD | | | Sediment | | | Flow | | | Nutrients | | | DCD×Sed | | DCD×Flow | | DCD×Nut | | Sed×Flow | | Sed×Nut | | Nut×Flow | | DCD×Sed×Nut | |
|---|---|---|---|---|---|---|---|---|---|---|---|---|---|---|---|---|---|---|---|---|---|---|---|---|---|---|---|
| **SPECIES LEVEL** | | ***P*** | **ES** | **dir.** | ***P*** | **ES** | **dir.** | ***P*** | **ES** | **dir.** | ***P*** | **ES** | **dir.** | ***P*** | **ES** | ***P*** | **ES** | ***P*** | **ES** | ***P*** | **ES** | ***P*** | **ES** | ***P*** | **ES** | ***P*** | **ES** |
| Taxon richness | | 0.108 | | | 0.661 | | | 0.549 | | | 0.386 | | | 0.286 | | 0.772 | | 0.191 | | 0.749 | | 0.826 | | 0.203 | | | |
| Simpsons evenness | | **<0.001** | 0.24 | + | 0.678 | | | 0.949 | | | 0.798 | | | 0.554 | | 0.264 | | 0.200 | | 0.694 | | 0.200 | | 0.919 | | | |
| | | | | | | | | | | | | | | | | | | | | | | | | | | | |
| *MANOVA* (species > 1 %, n=16) | | **<0.001** | 0.71 | | **<0.001** | 0.87 | | **<0.001** | 0.80 | | 0.680 | | | 0.099 | 0.46 | **0.039** | 0.51 | 0.381 | | **0.009** | 0.57 | 0.377 | | 0.256 | | | |
| *MANOVA* (species > 0.3 %, n=43) | | **0.054** | **0.96** | | **<0.001** | 1.00 | | **0.029** | 0.97 | | 0.240 | | | 0.539 | | 0.307 | | 0.169 | | 0.086 | 0.96 | 0.196 | | 0.414 | | | |
| **Abundant Species** | % | | | | | | | | | | | | | | | | | | | | | | | | | | |
| *Flavobacterium* UAS | 3.4 | **0.030** | 0.10 | + | 0.681 | | | 0.427 | | | 0.703 | | | 0.997 | | 0.508 | | 0.689 | | 0.924 | | 0.295 | | 0.784 | | | |
| *Pseudomonas* UAS 1 | 3.0 | **<0.001** | 0.41 | - | 0.820 | | | 0.926 | | | 0.272 | | | 0.390 | | 0.276 | | 0.237 | | 0.647 | | 0.361 | | 0.873 | | | |
| *Massilia varians* | 2.2 | **<0.001** | 0.29 | - | **<0.001** | 0.25 | - | 0.069 | 0.07 | + | 0.184 | | | 0.055 | 0.08 | 0.297 | | 0.060 | 0.07 | 0.526 | | 0.895 | | 0.250 | | | |
| *Rhodobacter* UAS | 2.0 | **<0.001** | 0.27 | + | **0.006** | 0.15 | - | 0.061 | 0.07 | - | 0.638 | | | 0.083 | 0.06 | 0.174 | | 0.476 | | 0.535 | | 0.651 | | 0.731 | | | |
| *Luteolibacter* UAS | 1.8 | **<0.001** | 0.36 | + | 0.068 | 0.07 | - | **0.002** | 0.18 | - | 0.719 | | | 0.311 | | **<0.001** | 0.21 | 0.098 | 0.06 | 0.708 | | 0.364 | | 0.066 | 0.07 | | |
| *Exiguobacterium sibiricum* | 1.8 | **<0.001** | 0.24 | - | **0.009** | 0.14 | + | 0.084 | 0.06 | - | 0.061 | 0.07 | + | 0.058 | 0.07 | 0.091 | 0.06 | 0.128 | | 0.119 | | 0.265 | | 0.718 | | | |
| *Pseudomonas* UAS 2 | 1.8 | 0.251 | | | 0.449 | | | 0.315 | | | 0.501 | | | 0.979 | | 0.784 | | 0.568 | | 0.755 | | 0.215 | | 0.518 | | | |
| *Phormidium* UAS | 1.7 | 0.152 | | | **0.002** | 0.18 | + | 0.359 | | | 0.292 | | | 0.913 | | 0.105 | | 0.628 | | 0.516 | | 0.286 | | 0.235 | | | |
| *Pseudomonas* UAS 3 | 1.5 | **<0.001** | 0.41 | - | 0.636 | | | 0.672 | | | 0.271 | | | 0.450 | | 0.434 | | 0.329 | | 0.810 | | 0.635 | | 0.506 | | | |
| *Acinetobacter* UAS | 1.4 | **<0.001** | 0.29 | - | **0.007** | 0.14 | - | 0.368 | | | 0.724 | | | **0.024** | 0.10 | 0.175 | | 0.514 | | 0.851 | | 0.819 | | 0.216 | | | |
| *Erwinia billingiae* | 1.3 | **<0.001** | 0.30 | - | **0.022** | 0.11 | - | **0.001** | 0.20 | - | 0.359 | | | 0.130 | | **0.011** | 0.13 | 0.958 | | 0.382 | | 0.984 | | 0.828 | | | |
| *Novosphingobium* UAS | 1.3 | **0.014** | 0.12 | + | **<0.001** | 0.21 | + | 0.121 | | | 0.253 | | | 0.205 | | 0.254 | | 0.922 | | 0.149 | | 0.072 | 0.07 | 0.725 | | | |
| *Pedobacter* UAS | 1.3 | **0.027** | 0.10 | + | 0.455 | | | 0.588 | | | 0.547 | | | 0.699 | | 0.759 | | 0.854 | | 0.965 | | 0.309 | | 0.589 | | | |
| *Janthinobacterium lividum* | 1.3 | **<0.001** | 0.33 | - | 0.722 | | | 0.680 | | | 0.339 | | | 0.803 | | 0.496 | | 0.603 | | 0.951 | | 0.756 | | 0.807 | | | |
| Comamonadaceae UAS | 1.1 | **0.022** | 0.11 | + | **0.021** | 0.11 | + | **<0.001** | 0.46 | - | 0.421 | | | 0.889 | | 0.093 | 0.06 | 0.287 | | 0.116 | | 0.657 | | 0.225 | | | |
| *Sphingopyxis* UAS | 1.0 | **<0.001** | 0.38 | + | 0.257 | | | 0.350 | | | 0.967 | | | 0.925 | | 0.134 | | 0.447 | | 0.643 | | 0.202 | | 0.857 | | | |
| | 28.0% | | | | | | | | | | | | | | | | | | | | | | | | | | |
| | | | | | | | | | | | | | | | | | | | | | | | | | | | |
| **Common Species** | | | | | | | | | | | | | | | | | | | | | | | | | | | |
| Micrococcaceae UAS | 0.9 | 0.194 | | | **0.008** | 0.14 | + | **0.016** | 0.12 | + | 0.968 | | | 0.486 | | 0.190 | | 0.763 | | 0.999 | | 0.759 | | 0.637 | | | |
| ***Janthinobacterium* UAS 1** | 0.9 | **<0.001** | 0.29 | - | 0.530 | | | **<0.001** | 0.21 | + | 0.115 | | | 0.521 | | 0.152 | | 0.150 | | 0.662 | | 0.390 | | 0.178 | | | |
| **Enterobacteriaceae UAS 1** | 0.8 | **<0.001** | 0.32 | - | 0.203 | | | **0.035** | 0.09 | - | 0.661 | | | 0.325 | | **0.026** | 0.10 | 0.862 | | 0.847 | | 0.686 | | 0.728 | | | |
| *Zymomonas* UAS | 0.6 | **0.006** | 0.15 | + | **0.006** | 0.15 | - | 0.138 | | | 0.780 | | | **0.048** | 0.08 | 0.352 | | 0.777 | | 0.119 | | 0.637 | | 0.852 | | | |
| Xanthomonadaceae UAS | 0.6 | **<0.001** | 0.37 | + | 0.545 | | | 0.328 | | | 0.770 | | | 0.793 | | 0.300 | | 0.856 | | 0.257 | | 0.427 | | 0.798 | | | |
| **Caulobacteraceae UAS 1** | 0.6 | 0.059 | 0.07 | | 0.966 | | | 0.178 | | | 0.613 | | | 0.853 | | 0.523 | | 0.578 | | 0.924 | | 0.683 | | 0.606 | | | |
| ***Acinetobacter* UAS 2** | 0.5 | **<0.001** | 0.28 | - | **0.033** | 0.09 | - | 0.683 | | | 0.833 | | | 0.217 | | 0.468 | | 0.388 | | 0.115 | | 0.503 | | 0.287 | | | |
| ***Rhodobacter* UAS 2** | 0.5 | **<0.001** | 0.23 | + | **<0.001** | 0.30 | + | **<0.001** | 0.51 | - | 0.478 | | | 0.491 | | 0.058 | 0.07 | 0.836 | | 0.215 | | 0.777 | | 0.760 | | | |
| *Kaistobacter* UAS | 0.5 | **<0.001** | 0.41 | + | **<0.001** | 0.23 | + | 0.283 | | | 0.765 | | | 0.626 | | 0.207 | | 0.148 | | 0.869 | | 0.429 | | 0.831 | | **0.040** | 0.09 |
| *Sphingomonas echinoides* | 0.5 | **0.023** | 0.10 | + | 0.503 | | | 0.325 | | | 0.216 | | | 0.205 | | 0.550 | | 0.378 | | 0.221 | | 0.378 | | 0.262 | | | |
| SC-I-84 UAS | 0.5 | **<0.001** | 0.40 | + | 0.981 | | | 0.057 | 0.07 | | 0.814 | | | 0.985 | | 0.395 | | 0.858 | | 0.288 | | 0.962 | | 0.526 | | | |
| **Oxalobacteraceae UAS** | 0.5 | **<0.001** | 0.26 | - | 0.528 | | | **0.021** | 0.11 | + | 0.145 | | | 0.443 | | 0.092 | 0.06 | 0.101 | | 0.331 | | 0.198 | | 0.051 | 0.08 | | |
| ***Pseudomonas* UAS 4** | 0.5 | **<0.001** | 0.44 | - | 0.524 | | | 0.989 | | | 0.206 | | | 0.299 | | 0.301 | | 0.461 | | 0.846 | | 0.626 | | 0.543 | | | |
| Chitinophagaceae UAS 1 | 0.4 | **<0.001** | 0.26 | + | **0.001** | 0.20 | + | 0.778 | | | 0.750 | | | 0.526 | | 0.401 | | 0.771 | | 0.508 | | 0.523 | | 0.312 | | | |
| *Flavobacterium* UAS 2 | 0.4 | **0.014** | 0.12 | - | **0.018** | 0.11 | + | **<0.001** | 0.29 | + | 0.080 | 0.06 | - | **0.043** | 0.08 | 0.651 | | 0.669 | | 0.782 | | 0.631 | | 0.111 | | | |
| Pirellulaceae UAS 1 | 0.4 | **<0.001** | 0.41 | + | **<0.001** | 0.45 | - | 0.100 | 0.06 | | 0.156 | | | **0.049** | 0.08 | 0.945 | | 0.451 | | 0.753 | | 0.553 | | 0.269 | | | |
| **Caulobacteraceae UAS 2** | 0.4 | **0.014** | 0.12 | - | **0.004** | 0.17 | - | 0.131 | | | 0.531 | | | 0.241 | | 0.717 | | 0.237 | | 0.651 | | 0.988 | | 0.772 | | | |
| *Flavobacterium* UAS 3 | 0.4 | **0.002** | 0.19 | - | **<0.001** | 0.27 | + | **<0.001** | 0.28 | + | 0.763 | | | 0.122 | | 0.086 | 0.06 | 0.859 | | **0.046** | 0.08 | 0.821 | | 0.938 | | | |
| ***Rhodobacter* UAS 3** | 0.4 | **<0.001** | 0.29 | + | 0.368 | | | 0.170 | | | 0.876 | | | 0.671 | | 0.299 | | 0.572 | | 0.281 | | 0.828 | | 0.391 | | | |
| *Synechococcus* UAS | 0.4 | **<0.001** | 0.28 | + | **<0.001** | 0.55 | - | 0.300 | | | 0.658 | | | **0.002** | 0.19 | 0.319 | | 0.838 | | 0.590 | | 0.869 | | 0.283 | | | |
| **Enterobacteriaceae UAS 2** | 0.4 | **<0.001** | 0.33 | - | **0.007** | 0.14 | - | **<0.001** | 0.25 | - | 0.506 | | | 0.094 | 0.06 | **<0.001** | 0.22 | 0.796 | | 0.198 | | 0.742 | | 0.765 | | | |
| *Sphingomonas* UAS | 0.3 | 0.110 | | | **0.012** | 0.13 | - | 0.170 | | | 0.564 | | | 0.137 | | 0.510 | | 0.975 | | 0.385 | | 0.850 | | 0.324 | | | |
| ***Acinetobacter* UAS 3** | 0.3 | **<0.001** | 0.23 | - | **0.005** | 0.15 | - | 0.852 | | | 0.790 | | | 0.062 | 0.07 | 0.331 | | 0.666 | | 0.748 | | 0.739 | | 0.211 | | | |
| Verrucomicrobiaceae UAS | 0.3 | **<0.001** | 0.43 | + | **<0.001** | 0.22 | - | **0.002** | 0.18 | - | 0.100 | 0.06 | + | 0.265 | | **0.035** | 0.09 | **0.038** | 0.09 | 0.630 | | 0.879 | | 0.820 | | | |
| **Enterobacteriaceae UAS 3** | 0.3 | **0.006** | 0.15 | - | **<0.001** | 0.25 | - | 0.626 | | | **<0.001** | 0.37 | + | 0.123 | | 0.899 | | 0.068 | 0.07 | 0.923 | | **0.002** | 0.19 | 0.233 | | | |
| *Luteolibacter* UAS 2 | 0.3 | **0.008** | 0.14 | + | **<0.001** | 0.21 | + | 0.089 | 0.06 | | 0.061 | 0.07 | - | 0.899 | | **0.034** | 0.09 | **0.024** | 0.10 | 0.825 | | 0.989 | | 0.067 | 0.07 | | |
| ***Janthinobacterium* UAS 2** | 0.3 | **<0.001** | 0.28 | - | **0.003** | 0.17 | + | **0.001** | 0.21 | + | 0.196 | | | 0.214 | | 0.269 | | 0.356 | | 0.518 | | 0.196 | | 0.465 | | | |
| | 40.8% | | | | | | | | | | | | | | | | | | | | | | | | | | |

## 3. Descriptions of significant interactions

ABUNDANT PHYLA (>1.0% of all sequences)

DCD×sediment: Proteobacteria showed a negative, antagonistic DCD by sediment interaction (i.e. the combined effect of both stressors was less negative than predicted additively) (Fig. S1).

DCD×flow velocity: DCD had a stronger positive effect on the relative abundance of Verrucomicrobia at fast flow velocity than at reduced velocity (Fig. S1).

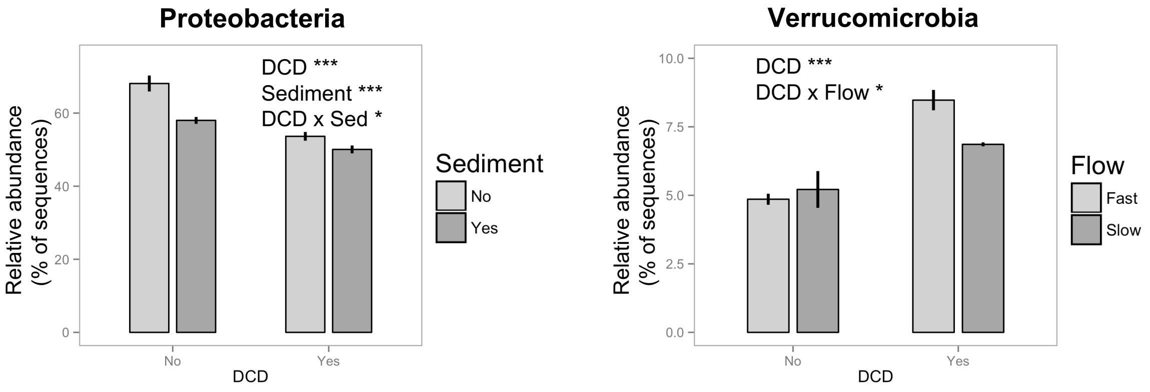


**Figure S1.** Mean relative abundances (± SE) of the abundant phyla that showed significant DCD by sediment (Proteobacteria) or DCD by flow velocity interactions (Verrucomicrobia). $P < 0.001$ = ***, $P < 0.01$ = **, $P < 0.05$ = *.

ABUNDANT ORDERS (>1.0% of all sequences)

DCD×flow velocity: DCD had a stronger positive effect on Verrucomicrobiales and Legionellales at fast flow velocity than at reduced velocity (Fig. S2). Enterobacteriales showed a double-negative, antagonistic response to DCD and reduced flow velocity, with the combined effect of both stressors being less negative than predicted additively (Fig. S2).

DCD×sediment: Sediment addition weakened the positive effect of DCD on the relative abundances of Pirellulales and Legionellales (Fig. S2).

Nutrients×flow velocity: Reduced flow velocity increased the relative abundance of Cytophagales mainly in mesocosms where nutrients were not enriched (Fig. S2).

Sediment×flow velocity: Sediment addition increased the relative abundance of WD2101 (Phycisphaerae) in mesocosms with faster flow velocity, but this pattern was reversed at reduced velocity (Fig. S2).

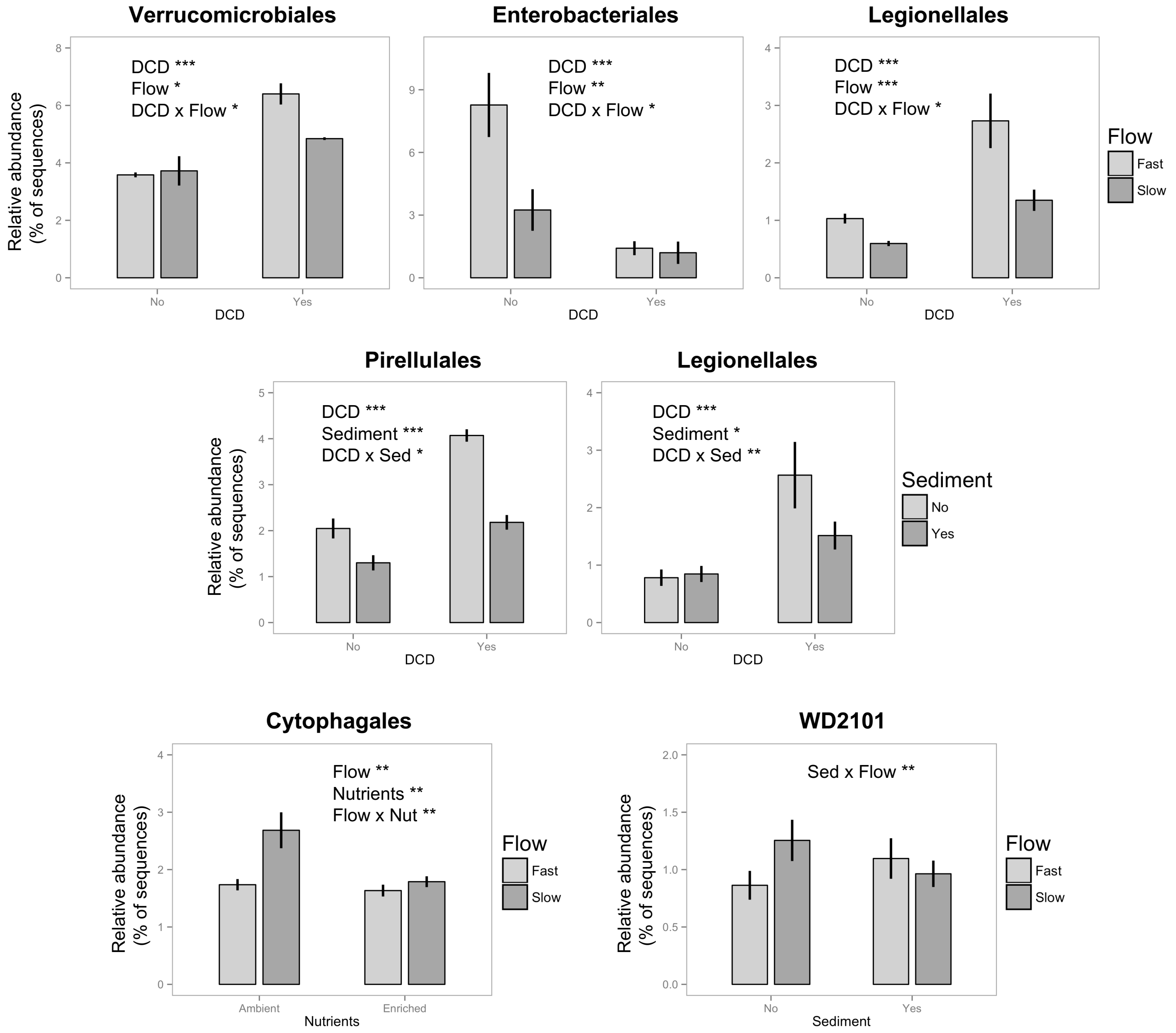


**Figure S2.** Mean relative abundances (± SE) of the abundant orders that showed significant DCD by flow velocity (Verrucomicrobiales, Enterobacteriales, Legionellales), DCD by sediment (Pirellulales, Legionellales), flow velocity by nutrient (Cytophagales), or sediment by flow velocity interactions (WD2101 Phycisphaerae). $P < 0.001$ = ***, $P < 0.01$ = **, $P < 0.05$ = *.

ABUNDANT GENERA (>1.0% of all sequences)

DCD×flow velocity: Reduced flow velocity weakened the positive effect of DCD on *Luteolibacter* and another unassigned genus of Verrucomicrobiaceae. *Erwinia* showed a double-negative, antagonistic response to DCD and reduced flow velocity, with the combined effect being less negative than predicted additively (Fig. S3).

DCD×sediment: Sediment addition decreased the positive effect of DCD on an unassigned genus of Pirellulaceae (Fig. S3).

Sediment×nutrients: Sediment addition had a stronger positive effect on *Novosphingobium* in mesocosms where nutrients were not enriched (Fig. S3).

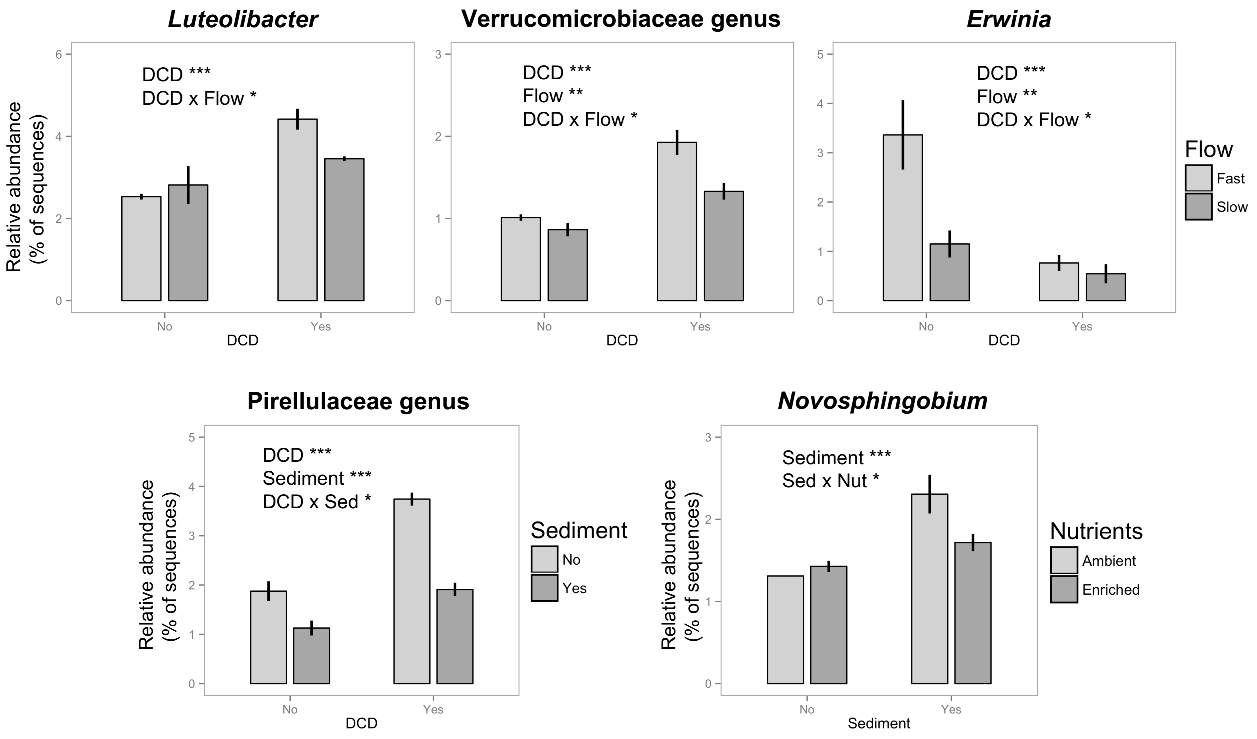


**Figure S3.** Mean relative abundances (± SE) of the abundant genera that showed significant DCD by flow velocity (*Luteolibacter*, an unassigned genus of the family Verrucomicrobiaceae, and *Erwinia*), DCD by sediment (a genus in the family Pirellulaceae), or sediment by nutrient interactions (*Novosphingobium*). $P < 0.001$ = ***, $P < 0.01$ = **, $P < 0.05$ = *.

ABUNDANT SPECIES (>1.0% of all sequences)

DCD×flow velocity: *Luteolibacter* sp. 1 responded more positively to added DCD at fast than at reduced flow velocity. *Erwinia billingiae* decreased with both DCD and reduced flow velocity in a negative antagonistic interaction (combined stressor effects less negative than predicted additively) (Fig. S4).

DCD×sediment: *Acinetobacter* sp. 1 also showed a double-negative, antagonistic response, but to DCD combined with sediment (Fig. S4).

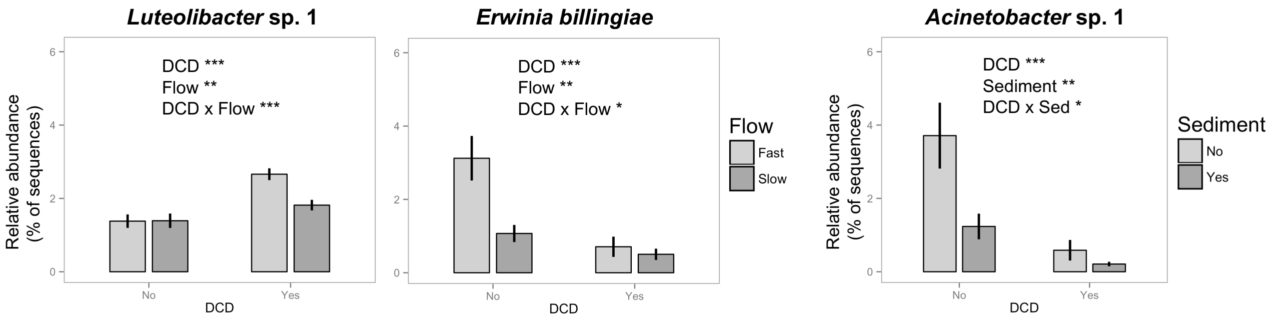


**Figure S4.** Mean relative abundances (± SE) of the abundant species that showed significant DCD by flow velocity (*Luteolibacter* species and *Erwinia billingiae*) or DCD by sediment interactions (*Acinetobacter* species). $P < 0.001$ = ***, $P < 0.01$ = **, $P < 0.05$ = *.

COMMON SPECIES (>0.3% of all sequences)

DCD×flow velocity: Two species of Enterobacteriaceae (sp. 1 and 2) showed double-negative, antagonistic responses to DCD and reduced flow velocity, with the combined effect of the two stressors being less negative than predicted additively (Fig. S5).

DCD×sediment: Sediment addition weakened the positive effect of DCD on a species of *Zymomonas*, a species of Pirellulaceae (sp. 1) and a species of *Synechococcus*. By contrast, sediment addition increased the relative abundance of *Flavobacterium* sp. 2 in mesocosms without DCD but not in those with DCD addition (Fig. S5).

Sediment×flow velocity: *Flavobacterium* sp. 3 showed a double-positive synergistic response to sediment addition and reduced flow velocity, with the combined effect being more positive than predicted additively (Fig. S5).

Sediment×nutrients: Enterobacteriaceae sp. 3 showed a greater increase with nutrient enrichment in mesocosms without sediment addition compared to those where sediment was added (Fig. S5).

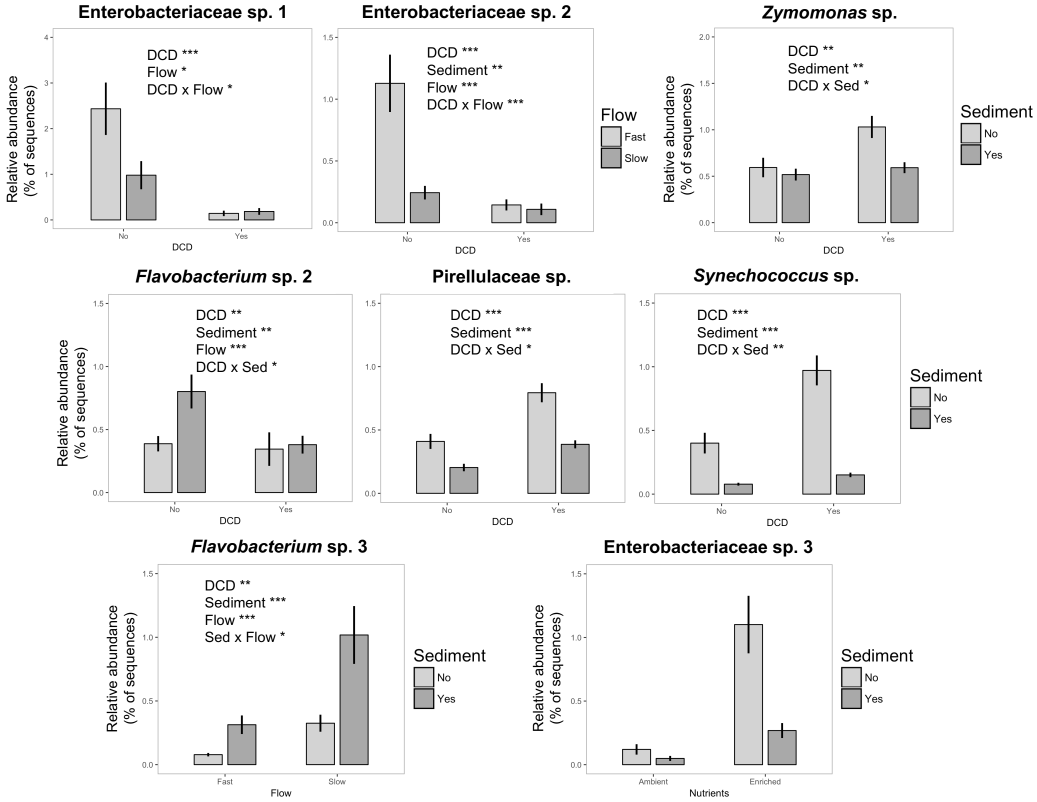


**Figure S5.** Mean relative abundances (± SE) of the abundant species that showed significant DCD by flow velocity (Enterobacteriaceae sp. 1 and 2), DCD by sediment (*Zymomonas* sp., *Flavobacterium* sp. 2, Pirellulaceae sp., *Synechococcus* sp.), flow velocity by sediment (*Flavobacterium* sp. 3) or sediment by nutrients interactions (Enterobacteriaceae sp. 3). $P < 0.001$ = ***, $P < 0.01$ = **, $P < 0.05$ = *.

DCD×nutrients and DCD×flow velocity: Verrucomicrobiaceae sp. and *Luteolibacter* sp. 2 showed greater increases with DCD addition in mesocosms with fast flow velocity compared to those where velocity was reduced. Verrucomicrobiaceae sp. also responded more positively to DCD when

nutrients were enriched as well, whereas *Luteolibacter* sp. 2 showed a negative response to nutrient enrichment in mesocosms without DCD but not when DCD was added (Fig. S6).

*Kaistobacter* sp. showed a positive synergistic response to DCD, sediment and nutrients, with abundances being highest when all three stressors were added (Fig. S6). This was the only significant 3-way interaction in our data.

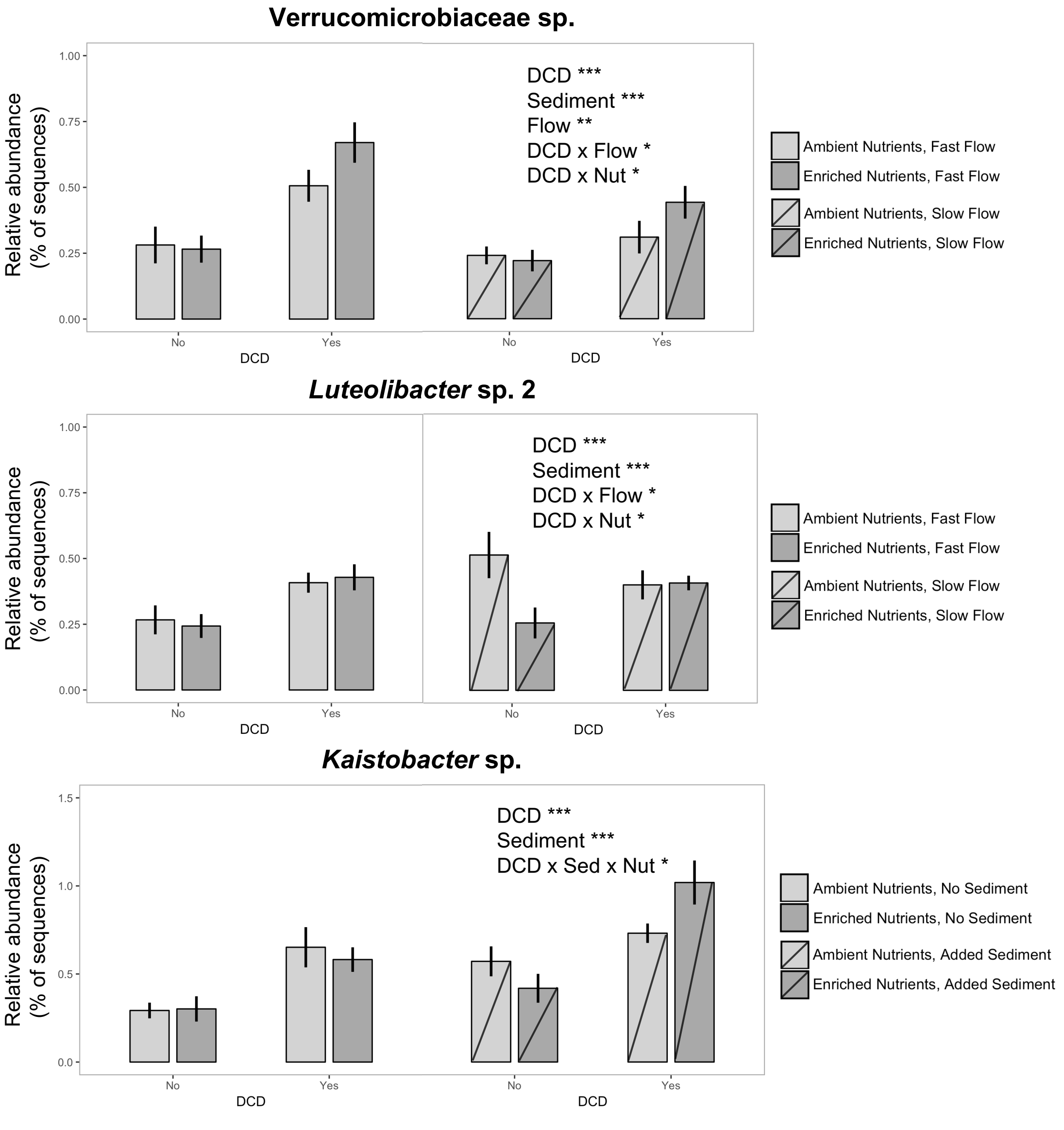


**Figure S6.** Mean relative abundances (± SE) of the abundant species that showed significant DCD by nutrients and DCD by flow velocity interactions (Verrucomicrobiaceae sp. and *Luteolibacter* sp. 2), and the only significant three-way interaction (DCD × sediment × nutrients; *Kaistobacter* sp.). $P < 0.001$ = ***, $P < 0.01$ = **, $P < 0.05$ = *.
